# Balancing stability and cellular interaction in surface-engineered small extracellular vesicles for pulmonary delivery

**DOI:** 10.1016/j.mtbio.2026.103637

**Published:** 2026-09-01

**Authors:** Maria José Sanchez, Pablo Leivar, Soraia Pinto, Helena Almeida, Margalida Esmeralda Artigues, Bruno Sarmento, Martí Lecina, Cristina Fornaguera

**Affiliations:** a Institut Químic de Sarrià (IQS), Universitat Ramon Llull (URL), Barcelona, 08017, Spain; b Laboratory of Biochemistry, Institut Químic de Sarrià (IQS), Universitat Ramon Llull (URL), Barcelona, 08017, Spain; c i3S – Instituto de Investigação e Inovação em Saúde, Universidade do Porto, Rua Alfredo Allen 208, Porto, 4200-135, Portugal; d Grup d’Electroquímica i Bioanàlisi (EQBA), Institut Químic de Sarrià (IQS), Universitat Ramon Llull (URL), Barcelona, 08017, Spain; e IUCS – CESPU – Instituto Universitário de Ciências da Saúde, Gandra, Portugal

**Keywords:** 3D lung model, Mucus penetration, Pulmonary drug delivery, Small extracellular vesicles, Surface engineering

## Abstract

Small extracellular vesicles (sEVs) are emerging as promising nanocarriers for non-invasive pulmonary drug delivery, yet their effectiveness is limited by sequential barriers imposed by airway mucus and the pulmonary epithelium-endothelium interface. Here, we investigated how post-secretory surface engineering with exogenous phospholipids modulates sEVs transport across lung-relevant barriers. eGFP-CD81 engineered sEVs were post-secretory modified at their surface composition using different lipid-to-vesicle ratios of either the zwitterionic lipid 1,2- Dilauroyl-sn-glycero-3-phosphocholine (DLPC) or the PEGylated lipid 1,2-Distearoyl-sn-glycero-3-phosphoethanolamine (DSPE) -PEG(5000) - Azide (DPA). Both modifications enabled controlled tuning of surface charge and colloidal stability without altering vesicle size at optimized lipid-to-vesicle ratios, although excessive DPA induced aggregation. The physicochemical properties and transport behaviour of engineered sEVs were evaluated in a reconstituted mucin gel and a 3D pulmonary epithelial-endothelial co-culture model. In mucin-containing medium, lipid-engineered sEVs showed enhanced diffusion compared with unmodified vesicles, with DLPC at a 30,000:1 ratio providing the highest cumulative permeability, sustained apparent permeability coefficients, and more diffusive motion profiles, as confirmed by multiple particle tracking. In the 3D co-culture, the same modification achieved the greatest cumulative permeability and basolateral accumulation, indicating efficient transcellular passage, while DPA-functionalized vesicles displayed moderate permeability and predominant retention at the apical epithelial layer. Through fluorescently-labelling EVs, we confirmed that all formulations were internalized by apical cells, but only DLPC-engineered sEVs reached detectable levels in basolateral cells. Neither DLPC nor DPA affected cell viability or barrier morphology. Overall, our results identify DLPC at 30,000:1 as a lead formulation that balances mucus penetration and efficient crossing of the pulmonary barrier, while DPA is better suited for applications requiring strong epithelial engagement. Our findings highlight rational phospholipid engineering as a powerful approach to tailor sEVs-based nanomedicines for pulmonary delivery.

## Highlights

• Small extracellular vesicles (sEVs) surface-engineering was achieved using either a zwitterionic (DLPC) or a PEGylated (DPA) lipid to fine-tune their physicochemical properties for pulmonary delivery.
• DPA functionalization reduced non-specific interactions and favored apical epithelial engagement, but was less efficient than DLPC in translocation.
• DLPC at 30,000:1 markedly improved sEVs transport across a mucin barrier, achieving the highest mucus permeability and most diffusive motion, superior transcellular passage and basolateral accumulation, while maintaining barrier integrity and cell viability.
• Rational phospholipid engineering of sEVs, evaluated in advanced lung-mimetic models, enables tailored design of vesicle-based nanocarriers for non-invasive pulmonary therapy.

## Graphical abstract

**Figure:**
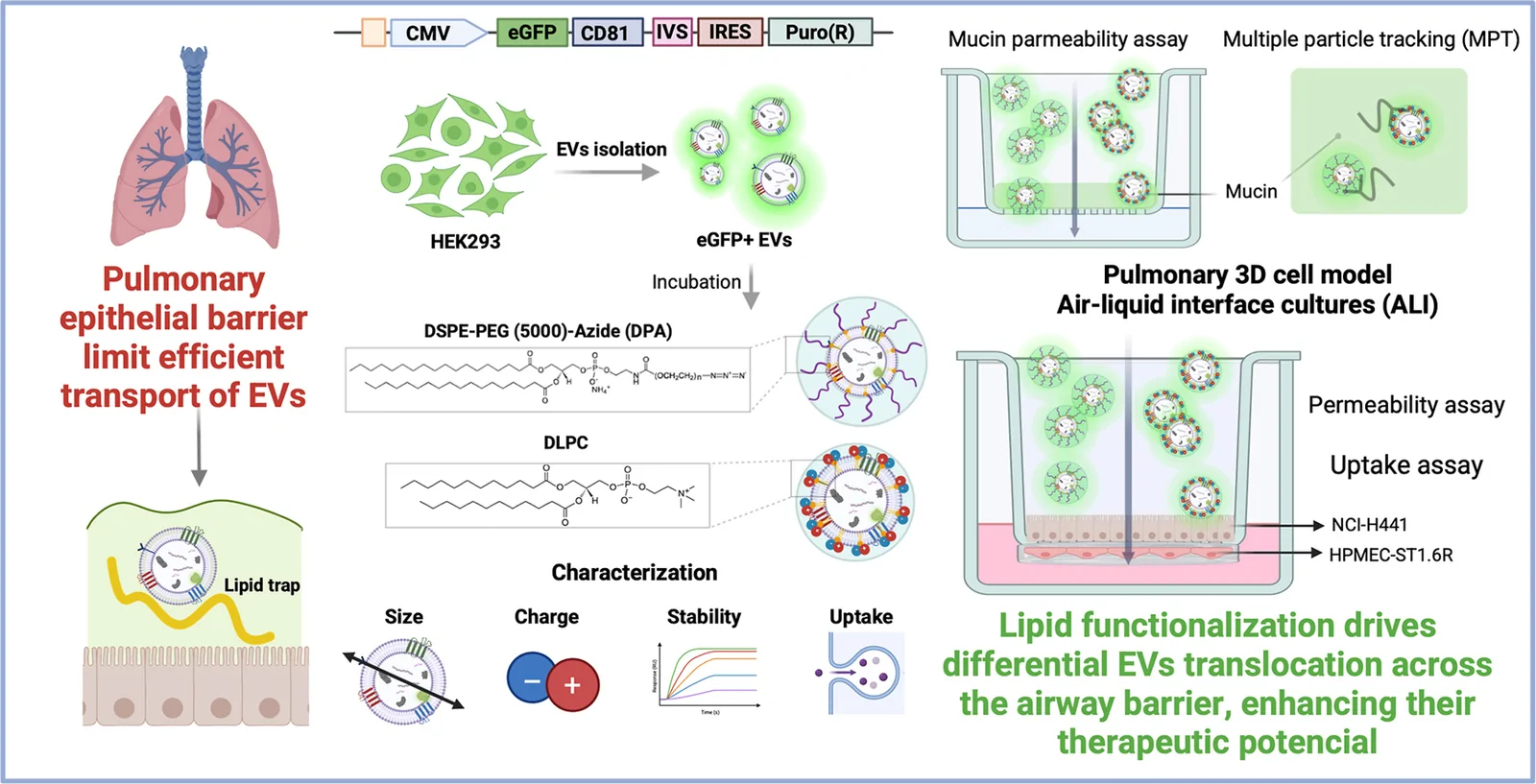

## 1 Introduction

The respiratory tract offers a highly attractive non-invasive route for drug administration, suitable for both local and systemic delivery. The lungs provide a very large absorptive surface area (≈120 m^2^), a dense capillary network, thin epithelial and endothelial layers, and relatively low proteolytic activity compared with the gastrointestinal tract [1,2]. Together, these features allow rapid onset of action, high bioavailability and avoidance of first-pass hepatic metabolism for a wide range of drugs. Beyond the treatment of primary lung disease (i.e. asthma, cystic fibrosis or chronic obstructive pulmonary diseases (COPD)), inhalation and intratracheal delivery are increasingly explored as alternatives to parenteral administration for systemic indications (i.e. vaccination or diabetes management), with the potential to improve patient adherence and enable self-administration [[3], [4], [5]].

However, efficient drug delivery to the lung remains challenging because inhaled nanosystems must overcome a series of multiple and sequential extracellular and cellular barriers before reaching their target cells [[3], [4], [5]]. In the conducting airways, the viscoelastic mucus layer traps particles and promotes their removal by the mucociliary clearance [[6], [7], [8]], while enzymes and antimicrobial compounds can destabilise delivery systems [9,10]. In distal regions, surfactant components, tight epithelial junctions, and alveolar macrophages further restrict transport across the air–blood barrier and accelerate clearance [[11], [12], [13]]. Together, these defence mechanisms greatly limit the fraction of an inhaled dose that can effectively reach the inner pulmonary tissue [7,14].

To address these barriers, pulmonary nanomedicines must be designed with physicochemical attributes that favour mucus penetration, epithelial interaction, and, when required, translocation across the air–blood barrier [[15], [16], [17]]. Among these features, surface properties are especially critical, as they govern colloidal stability, adhesive interactions with mucus, and cellular uptake [[18], [19], [20]]. In particular, a near-neutral or mildly negative, highly hydrated surface can reduce entrapment within mucus while preserving sufficient biointeractivity to enable epithelial engagement and transcytosis. For repeated administration, formulations must additionally maintain epithelial integrity and an acceptable safety profile [21,22]^,237,^ [23].

Nanomedicines can be rationally engineered to meet these requirements. In particular, small extracellular vesicles (sEVs) have emerged as highly promising pulmonary drug-delivery platforms. sEVs are cell-derived lipid bilayer vesicles, typically 30–150 nm in diameter, that naturally mediate intercellular communication by transporting proteins, lipids and nucleic acids between cells [24,25]. Their endogenous origin confers intrinsic biocompatibility, low immunogenicity and the capacity to exploit native trafficking and targeting pathways. Compared with purely synthetic nanocarriers, sEVs display complex membrane protein and glycan repertoires that can favour uptake by specific cell types or enhance barrier crossing [26,27]. These features have motivated intense efforts to develop EV-based therapeutic formulations across systemic and local routes, including to the respiratory tract [[28], [29], [30]], where nebulised or instilled EVs have already shown beneficial effects in models of lung injury and fibrosis [31,32]. In this context, recent advances in EV lyophilisation and the generation of dehydrated, more stable EV formulations may further support the development of inhalatory nanodelivery systems by improving storage, handling and formulation robustness, thereby facilitating their translational use in pulmonary administration [33]. Despite their promise, unmodified sEVs are not intrinsically optimized for mucus penetration. Native vesicles typically exhibit a net negative surface charge dominated by anionic phospholipids and exposed proteins [34]. Although this favours electrostatic repulsion from the likewise negatively charged mucin network, it does not fully prevent entrapment. Physical obstruction by the dense mucin mesh, transient ionic bridging mediated by divalent cations, and secondary interactions such as hydrogen bonding or hydrophobic contacts between vesicle membrane components and mucin glycans can all contribute to vesicle retention and rapid removal by mucociliary clearance [18,35,36]. In addition, interactions with pulmonary surfactant and uptake by resident immune cells further restrict vesicle access to the epithelial surface, ultimately limiting the effective delivery of sEVs cargo after administration [7,11,37].

Surface engineering therefore emerges as a key strategy to enhance sEV transport across the pulmonary barrier [38]. Traditionally, many mucus-penetrating nanomedicines have been coated with poly(ethylene glycol) (PEG) to create a hydrophilic stealth corona that minimises nonspecific protein and mucin adsorption, thereby reducing biofouling and prolonging residence time [20,39]. PEGylated lipids such as DSPE-PEG are widely used in clinically approved lipid nanoformulations, and similar strategies have been applied to EVs to improve colloidal stability and diffusion through mucosal tissues [40,41]. In parallel, zwitterionic materials are promising alternatives, including zwitterionic phospholipids and polymers bearing balanced positive and negative charges. Zwitterionic coatings exhibit ultra-low protein adsorption and strong hydration, leading to excellent resistance to biofouling, and have been shown to enhance nanoparticle transport across mucus and other mucosal barriers [[42], [43], [44]]. Short-chain zwitterionic lipids such as 1,2-dilauroyl-sn-glycero-3-phosphocholine (DLPC) can increase membrane fluidity and reduce electrostatic interactions with mucins, thereby favouring diffusion through the mucus mesh and facilitating fusion or uptake at epithelial surfaces [45]. At the same time, zwitterionic shells can limit recognition by opsonins and immune cells, potentially mitigating rapid clearance upon contact with pulmonary surfactant and resident macrophages, as suggested for zwitterionic polymer–conjugated biologics in lung delivery. Rational combinations of zwitterionic or PEG lipids may therefore offer a powerful toolkit to fine-tune sEV behaviour at each step of the pulmonary barrier [46,47].

Optimising these complex design parameters requires physiologically relevant *in vitro* models. Conventional two-dimensional submerged cultures of lung epithelial cell lines fail to reproduce key features of the airway microenvironment, such as mucus secretion, air-liquid interface (ALI) physiology, and multicellular crosstalk. Over the last decade, advanced three-dimensional systems, such as air–liquid interface cultures, have transformed our ability to study nanomedicines transport [[48], [49], [50], [51]]. These models can reproduce mucus production, surfactant secretion, and tight-junction formation. Such platforms are particularly well suited to dissecting the sequential journey of inhaled sEVs: mucus penetration, epithelial uptake, translocation across the air–blood barrier and interaction with endothelial and immune cells, and to benchmarking different surface-engineering strategies under near-physiological conditions [[48], [49], [50], [51]].

Within this context, the aim of this work was to harness these concepts (i.e. mucus penetration) to design a surface-engineered sEVs-based nanomedical platform capable of efficiently crossing the lung mucosa. Specifically, we focused on the incorporation of stealth-providing phospholipids, including zwitterionic and PEG components onto sEVs membranes to enhance mucus penetration and translocation across the pulmonary epithelium. We systematically characterized how these lipid modifications reshape the intrinsic and surface properties of sEVs, and we evaluate their transport, epithelial crossing and cell-type-specific uptake using advanced 3D *in vitro* models that recapitulate the mucus layer and the air–blood barrier [52]. Altogether, this study aims to establish a rational framework for pharmaceutically engineered, lung mucus penetrating sEVs formulations as non-invasive nanocarriers for pulmonary drug and gene delivery.

## 2 Experimental part

### 2.1 Material

The bicistronic plasmid C2-WT-171-176+pIRESpuro3 (GFP-CD81) was used as previously described (publication submitted). HEK293SF-3F6 cells were kindly provided by Dr. A. Kamen. BEAS-2B and NCI-H441 cells were obtained from ATCC (CRL-3588™; Manassas, USA). HPMEC-ST1.6R cells were kindly provided by Prof. C. James K (Johannes Gutenberg University of Mainz, Germany). Cell culture media included HyClone SFM4Transfx-293 (Cytiva), DMEM (Biowest), RPMI-1640, M − 199, and HBSS (Gibco/Thermo Fisher Scientific). Fetal bovine serum (FBS) was obtained from Lonza or PAN Biotech. Penicillin–streptomycin, L-glutamine, and Trypsin–EDTA were purchased from LabClinics, Biotecnomica Unipessoal, Thermo Fisher Scientific, or InnoProt, as indicated. The phospholipids DSPE-PEG(5000)-Azide (DPA, 880227P) and DLPC (850335P) were purchased from Avanti Polar Lipids. DMSO, DBCO-Cy5 (777374), DBCO-Cy3 (777366), Resazurin, Triton® X-100, formalin, ^Fluoromount^™, endothelial cell growth supplement (ECGS), heparin sodium salt, dexamethasone, and porcine gastric mucin type II were obtained from Sigma-Aldrich. ACO-600™ dye was purchased from ACORELA. Ultrafiltration was performed using Amicon® Ultra centrifugal filters (100 kDa MWCO, Millipore). Transwell® inserts (CellQART®), Matrigel™ (Corning), and standard tissue culture plasticware were used for cell-based assays.

### 2.2 Plasmid constructions

The bicistronic plasmid C2-WT-171-176+pIRESpuro3 (onward GFP-CD81) (6.61 kb) was used to generate the stable cell line expressing eGFP-CD81, as we described in our previous publication (publication submitted). In brief, it harbors a CMV promoter driving the eGFP-CD81 fusion, an internal ribosome entry site (IRES) upstream of the puromycin N-acetyl-transferase gene (PuroR) for selection in mammalian cells, an ampicillin resistance marker (AmpR) for propagation in *E. coli*, and a chimeric intron to enhance transcript levels. As a bicistronic construct, it enables co-expression of both eGFP-CD81 and puromycin resistance from a single mRNA, ensuring coordinated translation of both proteins, and the recovered sequence exactly matches the full reference with no mismatches. As a key advantage, this design allows robust generation of a fluorescent sEV-producing cell line, facilitating precise tracking of sEVs in subsequent experiments.

### 2.3 Cell lines

HEK293SF-3F6 (Human Embryonic Kidney Cells 239) (onwards HEK293) were maintained in HyClone SFM4Transfx-293 media (Cytiva) supplemented with 5% (v/v) Fetal Bovine Serum (FBS, Lonza), 1% (v/v) penicillin (100 U/ml) and streptomycin (100 μg/ml) (P/S, LabClinics) and 2 mM glutamine (LabClinics), as we described before in *Sanchez-Gomez* et al.*,* (under revision) including the sable modification of the cell line with the GFP expression plasmid cells (Human bronchial epithelial cells) (ATCC®, CRL-3588™) which were maintained in Dulbecco's Modified Eagle Medium (DMEM) (Biowest, L0102) supplemented with 2 mM L-glutamine, 1% (v/v) P/S and 5% (v/v) FBS at 37 °C in a humidified atmosphere containing 5% CO_2_. NCI-H441 (human lung adenocarcinoma cells) were obtained from ATCC® (Manassas, USA) and routinely cultured in T75 flasks (Sarstedt, 83.3911.002) using RPMI-1640 (Gibco, 21875-034) supplemented with 10% (v/v) FBS (PAN Biotech, P30-193031) and 1% (v/v) P/S (Biotecnomica Unipessoal, P06-07100). HPMEC-ST1.6R line (immortalized human pulmonary microvascular endothelial cells) was kindly provided by Professor C. James Kirkpatrick (Institute of Pathology, Johannes Gutenberg University of Mainz, Germany). They were maintained in T75 flasks and grown in M − 199 medium (Gibco, 31150030) further supplemented with 20% (v/v) FBS, 1% (v/v) P/S, 25 μg/mL endothelial cell growth supplement (ECGS) (Sigma Aldrich, E0760), 25 μg/mL heparin sodium salt (Sigma Aldrich, H3149) and 100 μg/mL L-glutamine (Thermo Scientific, 25030024).

For monolayer lines, medium was replaced every 2-3 days, and when cultures reached 70-80% confluence, cells were washed with 5 mL PBS, detached with Trypsin-EDTA (InnoProt, 0103) for 5 min at 37 °C, neutralized with complete medium, centrifuged, resuspended in fresh medium and subcultured into new T75 flasks. HEK293 cells, as suspension cell lines, were passaged 2 times per week, seeding at a 2.5 × 10^5^ cells/mL.

### 2.4 Methods

#### 2.4.1 sEVs isolation by differential centrifugation

HEK293 were seeded in Erlenmeyer using 25 mL of HyClone SFM4Transfx-293 media without FBS at 5x10^5^ cells/mL to produce fluorescent-labeled EVs. Every 3 days supernatant was collected, and cells subcultured again to 5x10^5^ cells/mL. Supernatant was preserved at −80 ◦C until isolation. Isolation of EVs from supernatant was performed by differential centrifugation, as we set up previously [53]^,^ using an Avanti-J30I high-speed centrifuge (Beckman Coulter, Germany). A first low-speed centrifugation step of 10,000 g at 4 ◦C for 45 min was performed to remove cells and large cell debris. The supernatant was then subjected to a second centrifugation step of 35,000 g at 4 ◦C for 70 min. With this second centrifugation step large debris and intact organelles were removed and EVs were recovered from the pellet. After removing the supernatant, 1/50 Phosphate Buffered Saline (PBS) of the initial sample volume was added to resuspend the pellet, concentrating then the EVs [53].

#### 2.4.2 sEVs surface modification

Phospholipid insertion was achieved via a passive, biocompatible process, inspired by the protocol described by *Warren* et al. [35], involving co-incubation of phospholipid molecules and sEVs. First, DSPE-PEG_5_-Azide (hereby DPA) (Avanti Research, 880227P) or DLPC (Avanti Research, 850335P) were dissolved in DMSO at 10 mg/mL each. An intermediate concentration, in aqueous stock solution, was generated where the lipid phase was then diluted with PBS (final volume 1 mL) by dropwise addition of PBS onto lipid phase, under continuous stirring at 37 °C, yielding final lipid concentrations of 0.01 mg/mL (16.08 μM DLPC and 1.71 μM DPA). Next, the required volume of sEVs stock suspension, previously quantified by nanoparticle tracking analysis (NTA), was added dropwise to the lipidic mixture with gentle agitation and incubated at 37 °C for 1 h under low-light conditions to form coated sEVs. Lipid molecules to EV particle of 50,000:1, 30,000:1, and 10,000:1 were tested. Two controls were included: 0:1 (fluorescent sEVs, unmodified) and 50,000:0 (phospholipid only, to evaluate possible micelle formation). Finally, non-incorporated lipids were removed by via ultrafiltration (UF) on a 100 kDa MWCO centrifugal filter Amicon® (Millipore, UFC5100). It consisted of a first centrifugation step at 14,000 g for 15 min, followed by two washing steps in PBS at the same speed. Finally, coated sEVs were recovered by inverting the filter and centrifuging at 1000 g for 1 min. The recovered volume was resuspended in enough PBS to reach the initial sample volume. To highlight, throughout the study, lipid:sEV ratios (e.g., 30,000:1) refer to the number of phospholipid molecules added per vesicle, as calculated from the lipid input and the sEV particle concentration determined by NTA.

#### 2.4.3 Fluorescently labelling of EVs

DSPE-PEG_5_-Azide (DPA) was utilized to enable a hydrophobically-inserted PEG coating on the sEVs surface. For studies involving characterization of DPA incorporation and stability, DPA was conjugated to the fluorescent moiety DBCO-Cy5 (Sigma-Aldrich, 777374) or DBCO-Cy3 (Sigma-Aldrich, 777366) via the azide-DBCO click chemistry reaction. DBCO-Cy5 or DBCO-Cy3 (1 mg/mL in DMSO (Sigma-Aldrich, 472301)) was added dropwise with continuous stirring to aqueous DPA stock for a 1:1 final molar ratio. This solution was then incubated for 6 h at RT with light shaking to facilitate the click reaction. After the click reaction, the solution containing DPA-Cy5 or DPA-Cy3 was added directly to sEVs samples for insertion. sEVs were stained with 1 μM and 2 μM ACO-600™ (ACORELA, Aco-600) dye and incubated at 37 °C for 1 h. ACO-600™ is a membrane-intercalating fluorescent dye that labels the vesicle lipid bilayer, allowing detection of membrane-associated sEV signal rather than luminal content. Following that, excess dye was removed by ultrafiltration: samples were centrifuged using the Amicon® Ultra-0.5 Centrifugal Filter Unit (100 kDa cutoff) at 3000 g for 10 min, the filter was washed twice by adding 400 μL of PBS and spinning again, after washing the filter was inverted and samples were recovered at 1000 g for 1 min, and stained samples were resuspended in PBS to the initial sample volume.

#### 2.4.4 Dynamic light scattering (DLS) for size measurement

The hydrodynamic size and particle‐size distribution by polydispersity index (PDI) of sEVs were characterized by dynamic light scattering (DLS) using a Malvern ZetaSizer Instrument (Malvern Instruments, United Kingdom, 4 mW laser) equipped with a He-Ne laser (λ = 633 nm) and a digital correlator, operated at a 173° scattering angle and at RT. sEVs samples were prepared by triplicated, resuspended in PBS and filtered through 0.22 μm membranes to obtain a concentration within the recommended measurement range (∼1·10^9^-particles/mL). Samples were thermally equilibrated for 10 min prior to acquiring 3 runs of 60 s each. PBS viscosity (η = 0.894 mPa s) and refractive index (n = 1.33) were considered for the analysis. In addition, zeta potential (ζ) was measured to assess vesicle surface charge and colloidal stability. sEVs were diluted 1:10 in Milli-Q® water adjusted to pH 8 (to mimic physiological ionic strength and deprotonate lipid head groups), equilibrated for 5 min, and analyzed by phase‐analysis light scattering under the same temperature conditions, electrophoretic mobility was converted to zeta potential via the Smoluchowski model.

#### 2.4.5 Nanoparticle tracking analysis (NTA) for size and concentration assessment

EVs size and concentration were determined via NTA using NanoSight NS300 equipped with NTA 3.4 analytical software. Briefly, samples were diluted in 0.22 μm filtered PBS and analyzed with light scatter mode using continuous flow rate and 60 s videos were recorded by triplicated per sample with a camera level of 11. Software settings for analysis were kept constant for all measurements (screen gain 10, detection threshold 10). For the detection of fluorescent eGFP particles Blue488 nm laser was used with camera level 16. Software settings for fluorescent EVs detection were changed and kept constant for all measurements (screen gain 10, detection threshold 3). The NTA measurement in flow mode were used to calculate the percentage of eGFP positive particles over the total number of scatter particles in the sample.

#### 2.4.6 Förster resonance energy transfer (FRET) for stability measurement

The stability and insertion of DPA-DBCO-Cy3 on the sEV surface were assessed by Förster resonance energy transfer (FRET) using the fluorescence mode of a microplate reader (Infinite M Plex microplate reader from TECAN). Approximately 2.50x10^9^ coated sEVs (200 μL/well, in triplicate) were dispensed into black-walled, clear-bottom 96-well plates (Merck, M5686). Samples were excited at 480 nm (eGFP excitation), and the donor emission peak of eGFP (∼510 nm) was used to indirectly excite the Cy3 acceptor (∼550 nm), Cy3 fluorescence was then recorded at 570 nm. Both excitation and emission bandwidths were set to 10 nm, with detector gain optimized to avoid saturation. Controls included, only PBS, unmodified sEVs (background) and wells containing free Cy3 (direct-excitation reference) at used concentrations subjected to the same conditions. Measurements were taken immediately and every 15 min up to 14 h at 37 °C to monitor complex stability.

#### 2.4.7 Flow cytometry for sEVs visualization with DPA-Cy5

Visualization of sEVs conjugated with DPA-Cy5 were performed with Beckman Coulter CytoFLEX S (Beckman Coulter, Germany). Conjugated vesicles were washed with PBS and recovered by ultrafiltration (Amicon® Ultra, 100 kDa) to remove unbound dye and resuspended in 0.22 μm-filtered PBS. Samples were diluted to an appropriate measurement concentration (≈1 × 10^9^ sEVs/mL) and loaded in triplicate. Triggering was set on the Cy5 fluorescence channel (638 nm laser; 660/10 nm emission filter) to improve detection of labeled particles, while VSSC (side scatter collected with the 405 nm laser) was recorded and plotted against Cy5 intensity (VSSC vs Cy5) to better discriminate sEVs from background. In addition to Cy5, the expressed eGFP signal of the sEVs was recorded (488 nm laser; 525/40 nm emission filter). Low flow rates were used to minimize coincident events (swarm), and a fixed acquisition endpoint was applied. Detector gain was optimized using controls, and spectral compensation was performed using single-stained controls. PBS only (blank), unlabeled sEVs (background), and the free DBCO-Cy5 fraction recovered from the wash (free-dye control) were included.

#### 2.4.8 Confocal scanning fluorescence microscopy (CSFM) for visualization of sEVs conjugated with DPA-Cy5

For CSFM visualization of sEVs samples, 10 μL of previously modified and purified suspensions at a concentration of 5 × 10^9^ sEVs/mL were deposited onto coverslips and allowed to dry for 15 min. Once completely dried, 10 μL of 10% (v/v) formalin (Sigma Aldrich, HT501128) were added and left to dry for an additional 15 min. The samples were then washed three times with PBS. Finally, coverslips were mounted on glass slides using Fluoromount™ mounting medium (Sigma Aldrich, F4680) and visualized by confocal scanning fluorescence microscopy (CSFM) using a Leica TCS SP8 laser-scanning confocal spectral microscope. Image analysis was performed with ImageJ software under identical parameters for all samples.

#### 2.4.9 LC-MS/MS (QTRAP®7500) for lipid quantification

The quantification of phospholipids (DPA and DLPC) incorporated onto the surface of sEVs was performed using a Triple Quad™ 7500 LC-MS/MS System-QTRAP®. In brief, surface-modified sEV samples were first purified by ultrafiltration to separate lipid-incorporated vesicles from non-incorporated phospholipids, and the resulting retentate and filtrate fractions were diluted in a solvent compatible with each analyte and adjusted to the calibration range prior to LC-MS/MS analysis. Samples were then analyzed by reverse-phase chromatography on a Kinetex C18 column coupled to tandem mass spectrometry operating in positive electrospray ionization and multiple reaction monitoring mode, using optimized precursor/product ion transitions for each phospholipid. Quantification was performed against external calibration curves prepared independently for DPA and DLPC, and the measured phospholipid amounts were finally normalized to the number of sEVs determined by NTA after the last purification step. For further details, a completed description of the method is presented in the SI, as **Explanation S1.**

#### 2.4.10 Cell viability assays

NCI-H441 and HPMEC-ST1.6R cells were seeded in 96-well plates (n = 3) at densities of 2 × 10^4^ and 1 × 10^4^ cells per well, respectively, and allowed to adhere and grow overnight at 37 °C in an incubator with 5% CO_2_. The following day, the medium was removed, and cells were incubated with different concentrations of surface modified sEVs samples, 2.50 × 10^10^ sEVs/cell, diluted in the respective complete medium for 24 h at 37 °C and 5% CO_2_. A negative control (cells incubated with 1% (v/v) Triton X-100 (Thermo Fisher Scientific, A16046) in medium), a positive control (cells incubated with medium only) and a blank (medium without cells) were included. After 24 h, 10% (v/v) Resazurin reagent (Sigma Aldrich, 199303) (diluted in medium) was added to each well and plates were incubated in dark conditions at 37 °C for 3 h in a humidified atmosphere with 5% CO_2_. Resazurin is reduced to resorufin, a highly fluorescent compound (excitation 530 nm, emission 590 nm), and fluorescence was measured using a microplate reader (SpectraMax iD3, Molecular Devices). Cell viability was calculated according to **Equation 1**, with the blank used to correct background signal.

$$Cell\;viability\;\left(\%\right)=\frac{Fluorescence\;signal\;of\;sample-Fluorescence\;signal\;of\;blank}{Fluorescence\;signal\;of\;positive\;control-Fluorescence\;of\;blank}\cdot 100$$

Equation 1: Equation for calculating cell viability (%).

#### 2.4.11 3D co-culture model of the air-blood barrier

Following the previously stablished protocol [52], a 12-well Transwell® insert (CellQART®, 9311012, 1.1 cm^2^, pore size 1.0 μm) was inverted on a well of 6 well-plate (Greiner, 657160) and the basolateral side was coated with 50 μL/cm^2^ of Matrigel™ (Corning, CLS354234) (protein concentration 4.0 mg/mL), followed by incubation for 1 h at 37 °C. HPMEC-ST1.6R cells were then seeded (5.0 × 10^4^ cells/Transwell®) on the basolateral side of the membrane and incubated for an additional 2 h to allow cell adhesion. Then, the Transwell® was inverted again and placed into the well. Next, 1.0 × 10^5^ NCI-H441 cells were seeded on the apical side of each Transwell®. The co-culture (NCI-H441 + HPMEC-ST1.6R) was incubated for 24 h in RPMI 1640 medium (0.5 mL apical/1.5 mL basolateral) supplemented with 5% (v/v) FBS, 1% (v/v) P/S, 25 μg/mL ECGS, 100 μg/mL L-glutamine and 25 μg/mL sodium heparin. On the following day the medium was aspirated and fresh complete medium, further supplemented with 200 nM dexamethasone (Sigma Aldrich, D4902) (to promote epithelial polarization) and 1% (v/v) Insulin-Transferrin-Selenium (ITS) (Thermo Fisher Scientific, 51500056), was added to each Transwell®, cells were kept under liquid-liquid conditions (LLC) for 48 h until day 3 of culture. Thereafter cells were transitioned to the air-liquid interface (ALI) by removing the apical medium and maintaining 0.5 mL of complete medium (including dexamethasone and ITS) in the basolateral compartment. Cells were kept under ALI for a further 24 h. On day 4 the medium was removed, and cells were washed with PBS to remove dexamethasone. The 3D co-culture was incubated for an additional 24 h (until day 5 of culture).

#### 2.4.12 Transepithelial electrical resistance (TEER) measurement of *in vitro* cell culture models

Transepithelial electrical resistance (TEER) was measured at different time points during culture to evaluate barrier tightness. When cells were maintained under LLC, the electrode connected to an EVOM voltohmmeter (World Precision Instruments, Sarasota, USA) was placed at the lateral side of the Transwell® to record electric resistance. For ALI measurements, 0.5 mL and 1.0 mL of culture medium were first added to the apical and basolateral compartments, respectively, and after 1 h incubation at 37 °C with 5% CO_2_ TEER was measured by placing the electrode on the Transwell® system. TEER of blank inserts (Transwell® without cells) was also recorded, and samples were corrected; accordingly, TEER values were calculated as Equation (2).

$$TEER\;\left(\Omega \cdot {cm}^{2}\right)=\left({TEER}_{sample}-{TEER}_{blank}\right)\cdot membrane\;area\;\left(cm^{2}\right)$$

Equation 2: Equation for calculation of TEER.

#### 2.4.13 Cell permeability assays

Surface modified sEVs permeability was investigated as described elsewhere [52,54]. A volume of 0.5 mL of surface-modified sEVs suspension (including unmodified sEVs as control), prepared at 2.5 × 10^10^ sEVs/mL (≈50,000 sEVs/cell), meaning 1 × 10^10^ fluorescent sEVs/mL (≈25,000 fluorescent sEVs/cell) in Hank's Balanced Salt Solution (HBSS) (Gibco, 14025100) (pH 7.4), were added to the apical chamber and the plates were shaken at 100 rpm at 37 °C. Aliquots of 200 μL were withdrawn from the basolateral chamber at 0.25, 0.50, 1, 1.5, 2, 3, 4, 6, 8, 10, 12 and 24 h and replaced with an equal volume of pre-warmed HBSS (pH 7.4) to maintain constant medium volume. The amount of sEVs permeated across cell monolayers was determined by fluorescence intensity. At the end of the assay, the remaining apical medium was collected to account for non-permeated sEVs, and cells from each insert were harvested and lysed with 1% (v/v) Triton® X-100. Fluorescence was measured in the apical, basolateral and cellular fractions to calculate the fluorescence balance across compartments.

Quantification of vesicle transport was carried out using the fluorescence emitted by labeled sEVs. To ensure that fluorescence values corresponded to intact vesicles, parallel quantifications were performed by NTA, and linear regressions between particle concentration (NTA) and fluorescence intensity (SpectraMax i3x Microplate reader) were established. These calibration curves were then used to extrapolate the concentrations of vesicles recovered in the different compartments. The corresponding calibration curves showed good linearity within the working concentration range used in the transport assays (Figs. S6 and S9; R^2^ = 0.9975 and 0.9993, respectively). Because surface modification could influence vesicle-associated fluorescence, fluorescence-based quantification was interpreted together with NTA/fluorescent NTA analyses of basolateral fractions to confirm recovery of intact labeled vesicles across conditions.

Cumulative permeability was calculated from the concentrations measured in the basolateral compartment (**Equation 3**), and apparent permeability coefficients (P_app_) were calculated using **Equation 4**, where ΔQ/Δt is the steady-state flux (particles/s), C_0_ is the initial sEVs concentration in the apical compartment (particles/mL), and A is the membrane area of the insert (cm^2^). P_app_ values were estimated using C_0_ values based on the total sEVs input. Experiments were carried out in sextuplicate (n = 6).

$$Cumulative\;permeability\;\left(\%\right)=\frac{\left(Total\;number\;of\;fluorescent\;sEVs\right)}{\left(Theoretical\;number\;of\;fluorescent\;sEVs\right)}\cdot 100$$

Equation 3: Equation for calculation of cumulative permeability (%).

$$P_{app}=\left(\frac{\Delta Q}{\Delta t}\right)\cdot \left(\frac{1}{A\cdot C_{0}}\right)$$

Equation 4: Equation for calculation of P_app_).

A mucin barrier cell-free variant of the permeability assay was performed using 24-well Transwell® inserts (CellQART®, 9321012; membrane area 0.3 cm^2^, pore size 1.0 μm). 200 μL of porcine gastric mucin, Type II (Sigma Aldrich, M2378) (20 mg/mL) was added to the apical chamber of each insert and allowed to equilibrate for 1 h at room temperature; thereafter 200 μL of sEVs (2.5 × 10^10^ sEVs/mL), meaning 1 × 10^10^ fluorescent sEVs/mL, were placed on top of the mucin layer and 0.6 mL HBSS (pH 7.4) was added to the basolateral compartment, and plates were incubated at 37 °C with shaking at 100 rpm. Basolateral aliquots (200 μL) were sampled following the same time points as the cellular assay, replacing each withdrawal with pre-warmed HBSS to maintain constant volume. All conditions were run in triplicate (n = 3). Permeation was quantified by measuring fluorescence intensity in apical, basolateral and mucin fractions and the same parameters were calculated as for the cellular assay (cumulative permeability and P_app_) using the established equations.

The integrity of sEVs (size distribution) recovered from the basolateral fractions was verified by NTA. Basolateral samples were diluted in 0.22 μm-filtered PBS to the optimal concentration recommended by the instrument manufacturer and analyzed in triplicate to obtain particle size distributions and concentrations. NTA in fluorescence mode was performed to confirm that the measured signal derived from labeled particles (and not from free dye).

#### 2.4.14 Multiple particle tracking (MPT) analysis

The analysis of sEVs transport by multiple particle tracking (MPT) was performed following the methodology described by *Gabriel* et al. [55]. Porcine gastric mucin was reconstituted in isotonic PBS (pH 7.4) at 20 mg/mL using a vortex mixer and allowed to equilibrate for 30 min at RT. In parallel, GFP + sEVs (surface-modified as indicated elsewhere) were prepared in PBS at 1.5 × 10^10^ fluorescent sEVs/mL. To obtain a mucus-like matrix at 10 mg/mL of mucin, equal volumes of the 20 mg/mL mucin stock and the sEV suspension were gently mixed (final sEV concentration 7.5 × 10^9^ fluorescent sEVs/mL). The mixture was incubated for 1 h at RT to allow equilibration. Subsequently, 65 μL of the mucus-sEVs mixture were transferred into a preassembled frame chamber mounted on a glass microscope slide (VWR, 631-0147) and sealed with a coverslip to prevent evaporation.

The diffusivity of fluorescent sEVs was evaluated by analyzing their trajectories. MPT videos were recorded using a Hamamatsu ORCA-Flash4.0 digital CMOS camera (Hamamatsu, Japan) mounted on a Leica DMI6000 inverted epifluorescence microscope (Wetzlar, Germany) equipped with a 63×1.30 NA GLYC immersion objective. Videos (512 × 512 pixels, 16-bit, 299 frames per video) were collected with LAS X software at a frame interval of 67 ms. Acquired video files (.lif format) were preprocessed through background subtraction in ImageJ and two-dimensional Cartesian coordinates of particle centroids were extracted at sub-pixel resolution using the MosaicSuite 2D/3D single particle tracking plugin developed by *Sbalzarini and Koumoutsakos* [56]. Particle centroid coordinates were exported as CSV files, and trajectory analysis was carried out with MPTHub software (v1.0.3), developed by Prof. Bruno Sarmento laboratory, to obtain trajectories and mean-squared displacement (MSD) values. Only trajectories containing at least 100 consecutive frames were included in the analysis, with approximately 100 nanoparticles analyzed per condition.

#### 2.4.15 *In vitro* cell uptake assays

*In vitro* uptake of sEVs was assessed by complementary flow cytometry, imaging flow cytometry, and confocal microscopy approaches in both 2D and 3D cell culture models. In brief, HEK293 and BEAS-2B cells, as well as 3D co-culture Transwell® models, were incubated with fluorescently labeled sEVs at defined particle-to-cell ratios and for the indicated exposure times. After incubation, cells were washed and, when required, detached, fixed, and processed separately according to the culture format. Uptake efficiency was quantified by conventional flow cytometry using appropriate fluorescence channels and standardized gating strategies to exclude debris and doublets and to define fluorescent-positive cell populations relative to unlabeled sEV and untreated-cell controls. In parallel, intracellular and spatial localization of sEV-associated fluorescence was evaluated by confocal laser-scanning microscopy following fixation, permeabilization, nuclear and/or cytoskeletal staining, and image acquisition under identical settings across conditions. In selected experiments, uptake was further characterized by imaging flow cytometry to combine quantitative fluorescence detection with single-cell image analysis and morphological gating. Data were analyzed using the corresponding instrument software and ImageJ/IDEAS®, and results are reported from at least three independent replicates. More details on the experimental procedure can be found in **Explanation S2,** from SI.

#### 2.4.16 Software and statistical analysis

Preprocessing MPT were performed in Fiji software (ImageJ distribution, NIH, USA) using the MosaicSuite 2D/3D plugin, particle centroid coordinates were exported as CSV files. Trajectory analysis was carried out with MPTHub software (v1.0.3). Graphics were performed using GraphPad Prism® software (GraphPad Software, San Diego, USA)**.** Microscopy images were processed using Fiji software (ImageJ distribution, NIH, USA). All figures and diagrams were created using BioRender **(**www.biorender.com**)**.

All data presented, unless otherwise stated, represent the mean value ± standard deviation (SD) of at least, a triplicate independent sample. Statistical analyses were performed using GraphPad Prism® 10. Differences among groups were analyzed using One-way or Two-way ANOVA, as appropriate for the experimental design. When only selected comparisons were of interest, Sidak's multiple-comparison test was applied; when all pairwise comparisons among groups were relevant, Tukey's multiple-comparison test was used. Differences were considered to be significant at a level of ns = not significant, * = *p* < 0.05, ** = *p* < 0.01, *** = *p* < 0.001 and ****p < 0.0001.

## 3 Results and discussion

### 3.1 Engineering sEVs surface for pulmonary delivery

Effective pulmonary delivery of sEVs requires surface engineering to improve stability of sEVs in the airways and modulates their transport across mucus and the pulmonary epithelium. Here, sEVs were functionalized with amphiphilic lipids that passively insert into the vesicle membrane, using DSPE-PEG_5_-azide (DPA) and 1,2-dilauroyl-phosphatidylcholine (DLPC) as representative PEGylated and zwitterionic/phosphatidylcholine lipids, respectively. DPA anchors via saturated C18 tails, forms a PEG corona that reduces adsorption of airway components and shifts surface charge, and provides a terminal azide for copper-free click conjugation of ligands [36,39,47]. DLPC, being a charge-neutral phosphatidylcholine, has been associated with increased membrane fluidity and reduced adhesive interactions in lipid systems, which might contribute to the improved transport behavior observed here [36,57,58]. Lipids were introduced by simple co-incubation with sEVs at 37 °C, enabling spontaneous insertion without any external energy input, and different lipid:EV ratios were compared to determine their impact on sEVs physicochemical properties and subsequent barrier-relevant behavior.

**Fig. 1:**
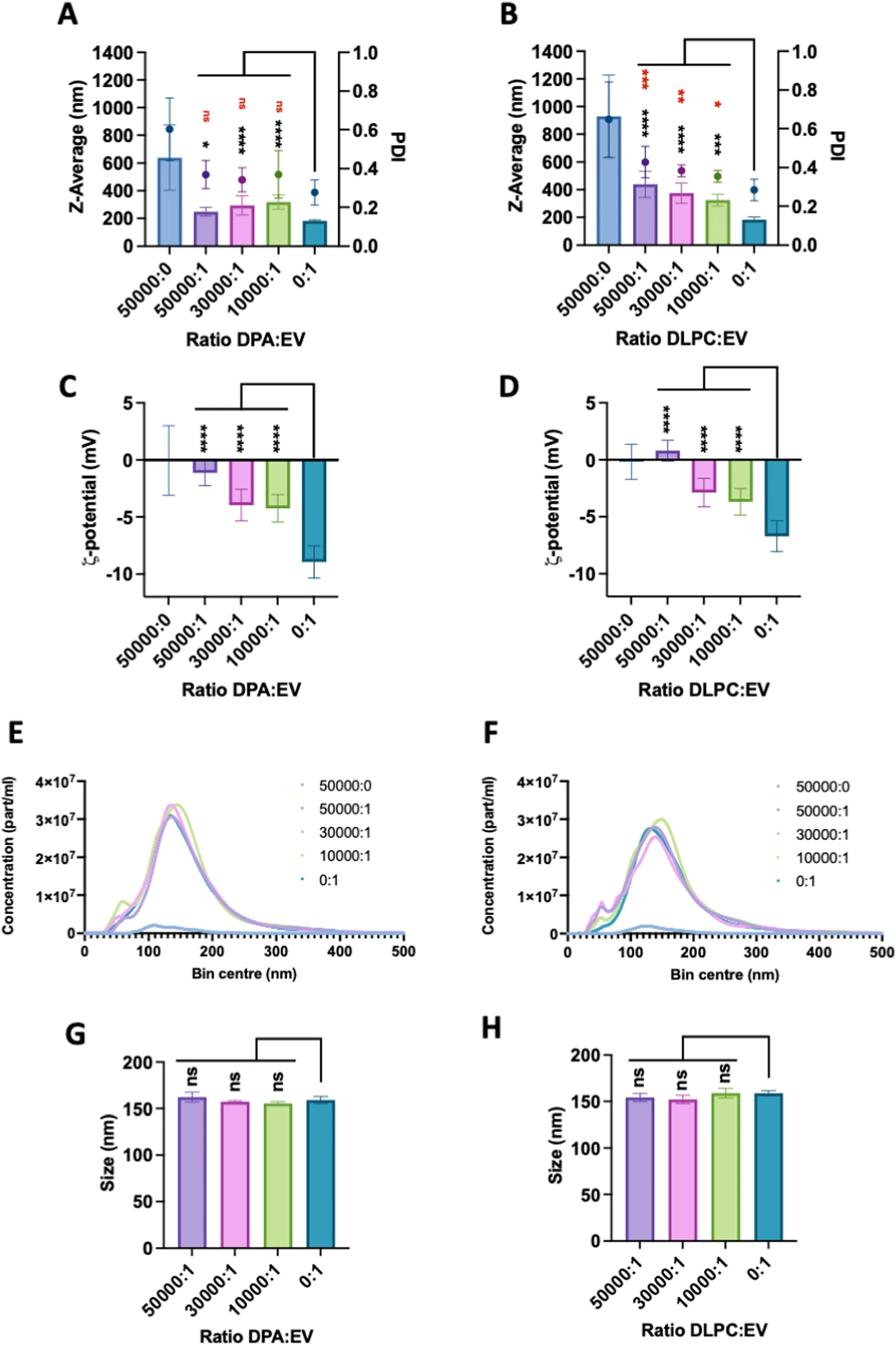
**Physicochemical characterization of sEVs modified with different DPA:EV or DLPC:EV ratios** (**A, B**) Z-average diameter (bars, left y-axis) and polydispersity index (PD,; points, right y-axis), and (**C, D**) ζ-potential (mV) of DPA and DLPC modified sEVs, respectively, by DLS; Particle concentration of (**E**) DPA-modified, (**F**) DLPC-modified sEVs. Particle size mean of (**G**) DPA-modified, (**H**) DLPC-modified sEVs. Ratios indicate the number of phospholipid molecules per vesicle. Controls include 0:1 (unmodified sEVs) and 50,000:0 (phospholipid only, used to assess micelle/aggregate formation). Data are presented as mean ± SD (n = 3). Black and red asterisks indicate statistical comparisons for Z-average diameter and PDI, respectively. Statistical analysis: ns = not significant, ****p < 0.0001, ***p < 0.001, **p < 0.01 and *p < 0.05.

After isolating CD81-eGFP engineered sEVs from HEK293 donor cells, lipid incorporation was first assessed by DLS (Z-average diameter, PDI, ζ-potential) and further validated by NTA (size and concentration). DLS showed an increase in Z-average diameter after modification with both lipids. For DPA (Fig. 1. A.), the largest size increases were observed at 30,000:1 and 10,000:1, whereas 50,000:1 yielded a smaller apparent diameter, consistent with saturation/crowding of PEG chains that adopt a more compact conformation and reduce the effective hydrodynamic footprint at very high DPA availability. In contrast, DLPC (Fig. 1B.) produced a clearer lipid-dose trend, with the largest diameters observed at 30,000:1 and 50,000:1, and smaller values at 10,000:1, consistent with stronger membrane rearrangements driven by phosphatidylcholine incorporation and increased bilayer fluidity.Phospholipid-only controls (50,000:0) displayed very large, highly variable diameters, consistent with lipid self-assembly into heterogeneous aggregates; these conditions were treated as aggregation controls rather than vesicle populations [[59], [60], [61]].

PDI values supported these trends in sample heterogeneity. DPA-modified sEVs showed consistently elevated PDI (∼0.4-0.5) across ratios compared with unmodified sEVs, suggesting increased heterogeneity, likely due to uneven accommodation of bulky DSPE-PEG in the intrinsically heterogeneous sEV membrane (Fig. 1A.). For DLPC, higher PDIs were observed at 50,000:1 and 30,000:1, while 10,000:1 yielded a lower PDI, indicating that excessive DLPC can increase heterogeneity (e.g. via lipid exchange or transient fusion-like events), whereas moderate insertion favors more homogeneous suspensions (Fig. 1B). As expected, phospholipid-only (50,000:0) controls showed the highest PDIs and variability, consistent with mixed aggregates.

ζ-potential was measured in diluted PBS (rather than ultrapure water) to ensure vesicle stability and a defined ionic environment for electrophoretic mobility [34,62]. Unmodified sEVs were negatively charged, consistent with anionic phospholipids and sialylated surface glycoconjugates. Both DPA and DLPC shifted ζ-potential toward neutrality across ratios (Fig. 1C **and D.)**: DPA likely shields surface charge via its PEG corona and exposed polar groups, while DLPC partially masks anionic surface components through its zwitterionic headgroup. In contrast, phospholipid-only controls showed unstable ζ values, in line with heterogeneous aggregates [34].

NTA provided single-particle, number-weighted confirmation that lipid insertion did not meaningfully alter vesicle recovery: DPA- and DLPC-functionalized samples showed concentrations comparable to unmodified sEVs and similar main population sizes (peak ∼120–150 nm) (Fig. 1E and F.). In the absence of sEVs (50,000:0), particle counts were near or below the method's sensitivity, consistent with formation of large aggregates (and possibly a minor fraction of small structures) that could not be robustly quantified, so these controls were excluded from size analysis. Fluorescently labeled sEVs intended for downstream assays showed equivalent physicochemical profiles (Fig. S1). The expected DLS-NTA differences were observed: DLS is intensity-weighted and can be influenced by hydration layers and aggregates, whereas (fluorescent) NTA is number-weighted and reflects the predominant vesicle population (Fig. 1G and H.). Overall, modified sEVs preserved their identity and core size without appreciable loss, while phospholipid-only conditions did not generate stable vesicle-like populations. Since diameter, PDI, and ζ-potential values were broadly comparable among some ratios, the selected formulations were chosen as stable representative conditions rather than based on a single physicochemical parameter. DPA 10,000:1 and 30,000:1 were selected to assess low-to-intermediate PEGylated lipid incorporation while avoiding the highest lipid input, whereas DLPC 30,000:1 and 50,000:1 were selected to evaluate intermediate-to-high phosphatidylcholine incorporation, both showing increased diameters consistent with membrane rearrangement.

Recent high-speed atomic force microscopy (HS-AFM) studies have provided complementary nanoscale evidence that sEVs display heterogeneous and dynamic surface organization under near-physiological conditions. In particular, HS-AFM videography has revealed distinct nanotopological features across sEV subpopulations and has shown that sEV morphology can respond sensitively to physicochemical perturbations [63,64]. In this context, the ratio-dependent changes observed here after DPA or DLPC insertion are consistent with the concept that surface lipid engineering can remodel the vesicle interfacial architecture, thereby influencing colloidal behavior and barrier transport performance.

### 3.2 Quantification of phospholipid incorporation onto sEVs

**Fig. 2:**
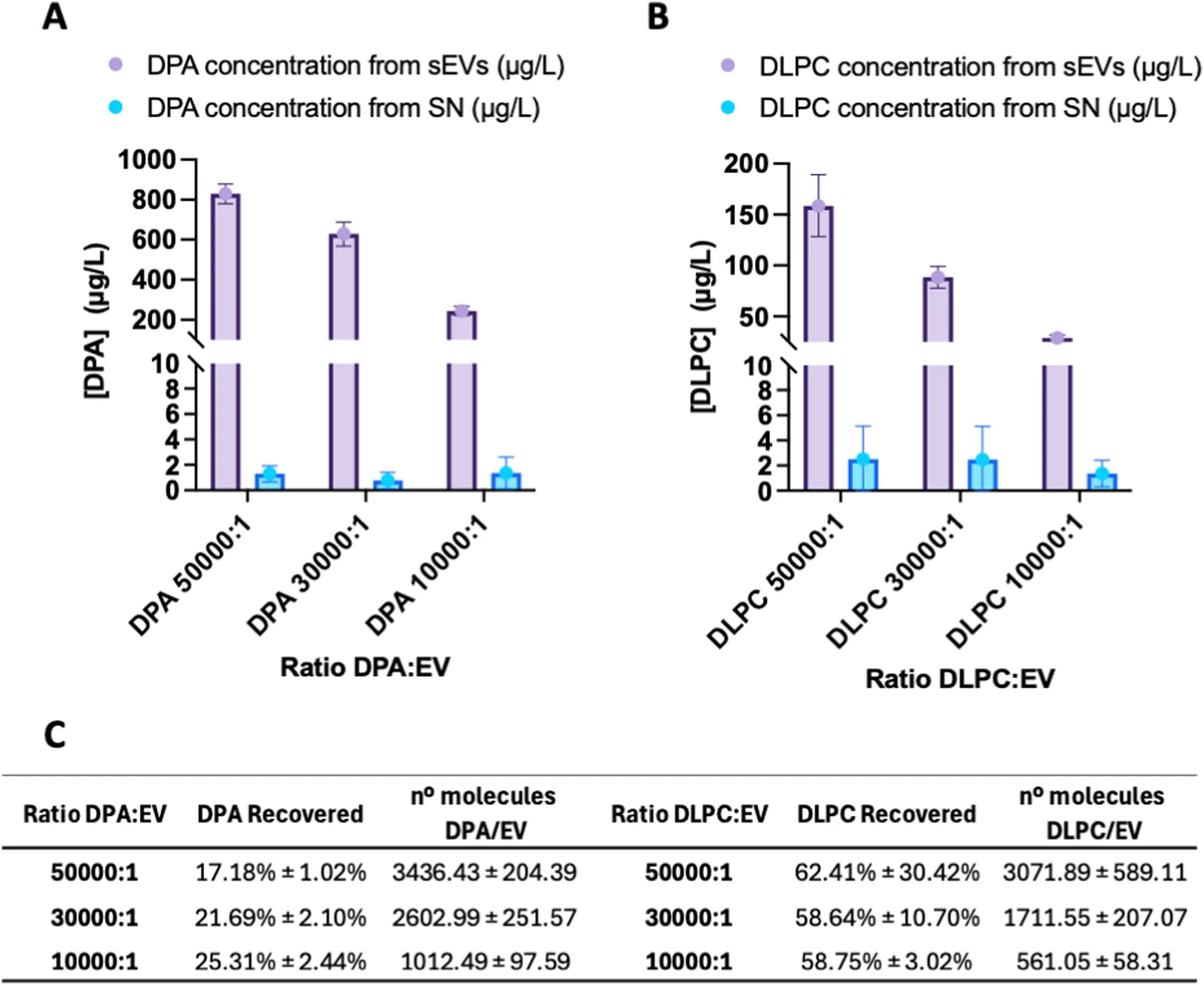
**Quantification of lipid incorporation into sEVs by QTRAP**®**.** Concentrations of (**A**) DPA and (**B**) DLPC detected in sEV retentates and supernatants at different ratios. (**C**) Quantification of recovery and number of molecules. Data are shown as mean ± SD (n = 3).

After confirming lipid incorporation onto sEVs surface from the changes in sEV physicochemical properties, phospholipid incorporation was quantified by targeted lipid analysis to estimate lipid-to-vesicle ratios and link these shifts to the degree of surface functionalization. Although DPA quantification was technically more challenging than DSPE, method optimization (**Explanation S3**, SI) enabled both DPA and DLPC to be quantified using a QTRAP® workflow. For DPA, the signal in the retentate increased with the input lipid:EV ratio (Fig. 2A.), indicating ratio-dependent insertion onto the sEV membrane and co-retention with vesicles during ultrafiltration. In contrast, supernatant (SN) levels were near-zero across conditions, consistent with free PEG-lipid being removed/diluted during washing and/or being retained/adsorbed by the filtration set-up rather than passing into the filtrate. Overall DPA recovery (retentate + SN) was low (∼17-25%; Fig. 2C.) and decreased with increasing input ratio, indicating that absolute quantification should be interpreted cautiously. This behavior is consistent with a combination of saturable insertion, adsorption/losses during ultrafiltration and washing, and analytical effects associated with this PEGylated lipid [65]. Accordingly, the estimated DPA molecules per sEV should be interpreted as a comparative approximation across the tested conditions rather than as an exact absolute measurement of surface composition. Even so, the estimates showed a clear ratio-dependent trend, increasing with feed ratio but with sub-linear scaling (e.g., the 50,000:1 condition rising less than expected/extrapolated from the 10,000:1 condition), supporting a saturation/steric crowding effect of bulky PEG chains. The resulting estimated surface densities fell within a physically plausible range for ∼120–150 nm vesicles, although these values should be considered approximate given the limited overall DPA recovery, corresponding to ∼1-5% of outer-leaflet lipids depending on ratio, within typical PEG-lipid ranges used before compromising membrane integrity or uptake [66,67].

DLPC displayed a similar distribution pattern but markedly higher efficiency. Most DLPC was recovered in the retentate with SN values close to zero, indicating effective membrane incorporation and/or retention of any non-inserted DLPC as aggregates large enough to be retained by the ultrafiltration membrane, with washes further lowering free lipid below detection (Fig. 2B.) [68]. In contrast to DPA, DLPC recovery was consistently high (∼58-62%; Fig. 2C.) across ratios, consistent with favorable bilayer integration driven by its phosphatidylcholine headgroup and short, fluid acyl chains. The calculated DLPC molecules per EV increased approximately proportionally with input ratio, translating to ∼0.8%, ∼2.5%, and ∼4.4% of estimated outer-leaflet lipids at 10,000:1, 30,000:1, and 50,000:1, respectively, with higher variability at the highest ratio. Overall, the near-linear scaling and higher recovery indicate that DLPC integrates onto sEV membranes more efficiently than DPA, likely because it lacks the steric constraints imposed by dense PEG coronas [69].

### 3.3 Click chemistry functionalization of DPA incorporated into the sEV membrane

After incorporating DPA, its azide group was leveraged for copper-free click labeling with a cyclooctyne-conjugated fluorophore (Cy5 or Cy3), restricting fluorescence to vesicles that effectively contain DPA and enabling direct comparison with the eGFP-CD81 reporter. The resulting DPA-dye conjugate was then inserted into sEV membranes using the same passive insertion protocol, generating a specifically labeled DPA^+^ sEV population. Incorporation was first assessed by nano-flow cytometry, discriminating eGFP^+^ vesicles (eGFP-CD81 expression) from Cy5^+^ vesicles (DPA-Cy5 insertion) and quantifying the Cy5^+^ fraction, with a phospholipid-only Cy5 control included to verify that Cy5 signal was not driven by free lipid aggregates.

**Fig. 3:**
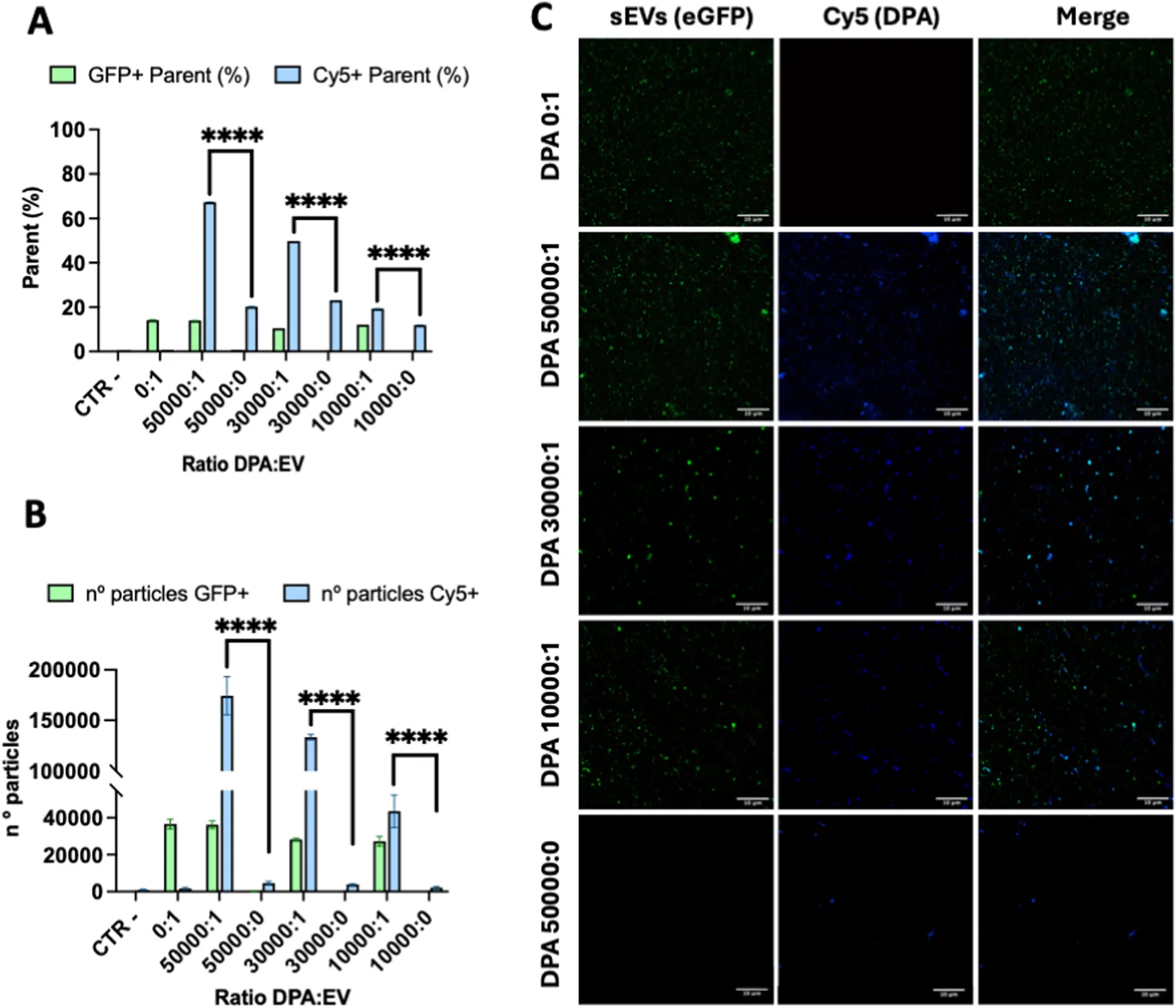
**Quantitative and qualitative analysis of sEVs labeled with DPA-Cy5 at different ratio**. (**A**) Percentage of +eGFP vesicles (intrinsic, genetically encoded) and +Cy5 vesicles (after DPA-Cy5 incorporation). (**B**) Absolute number of fluorescent particles detected for the same conditions, both by nanoflow cytometry. (**C**) Confocal microscopy images of sEVs modified with DPA-Cy5 at different ratios**.** eGFP signal (green) corresponds to genetically encoded vesicles, Cy5 signal (blue) indicates incorporation of DPA-Cy5, and merged images show colocalization. Objective 60x with 3x zoom. Scale bar = 10 μm. C- corresponds to the negative control of non-fluorescent vesicles, while the 50000:0, 30000:0 and 10000:0 ratio represent samples containing only phospholipid. Data are presented as mean ± SD (n = 3). Statistical analysis: ns = not significant, ****p < 0.0001, ***p < 0.001, **p < 0.01 and *p < 0.05.

Across lipid:EV ratios, the percentage of eGFP^+^ events remained constant, indicating that the click labeling and insertion steps did not affect the intrinsic eGFP signal (Fig. 3A and B.). Cy5^+^ detection was higher than eGFP^+^, consistent with DPA-Cy5 labeling a broader vesicle population, including vesicles carrying eGFP below the detection threshold and EVs without eGFP. Although the phospholipid-only Cy5 control showed a small Cy5^+^ background, vesicle-containing samples exhibited significantly higher Cy5^+^ counts, and absolute particle numbers confirmed that most of the Cy5 signal originated from DPA-Cy5 incorporated into vesicles rather than from residual lipid aggregates (supporting gating/plots in Figure S2). Overall, nano-flow cytometry supports efficient DPA-Cy5 functionalization without compromising eGFP reporting.

The same samples were then examined by confocal microscopy as an orthogonal qualitative check (Fig. 3C) [[70], [71], [72]]. Unmodified sEVs showed eGFP fluorescence without Cy5, while DPA-modified conditions displayed Cy5 signal that colocalized with eGFP, consistent with successful DPA-Cy5 insertion into vesicle membranes. At the highest ratio (50,000:1), larger Cy5-bright puncta were observed, and Cy5 signal was also detected in the phospholipid-only control, supporting the presence of lipid aggregates at excessive DPA loading. In contrast, lower/intermediate ratios (e.g., 10,000:1 and 30,000:1) showed a more dispersed Cy5 pattern consistent with a higher proportion of labeled vesicles and reduced aggregation. Together, these data indicate that DPA-Cy5 labeling is robust and compatible with eGFP-CD81 readout, while also revealing a practical upper limit in DPA loading beyond which sample homogeneity is compromised by aggregate formation.

**Fig. 4:**
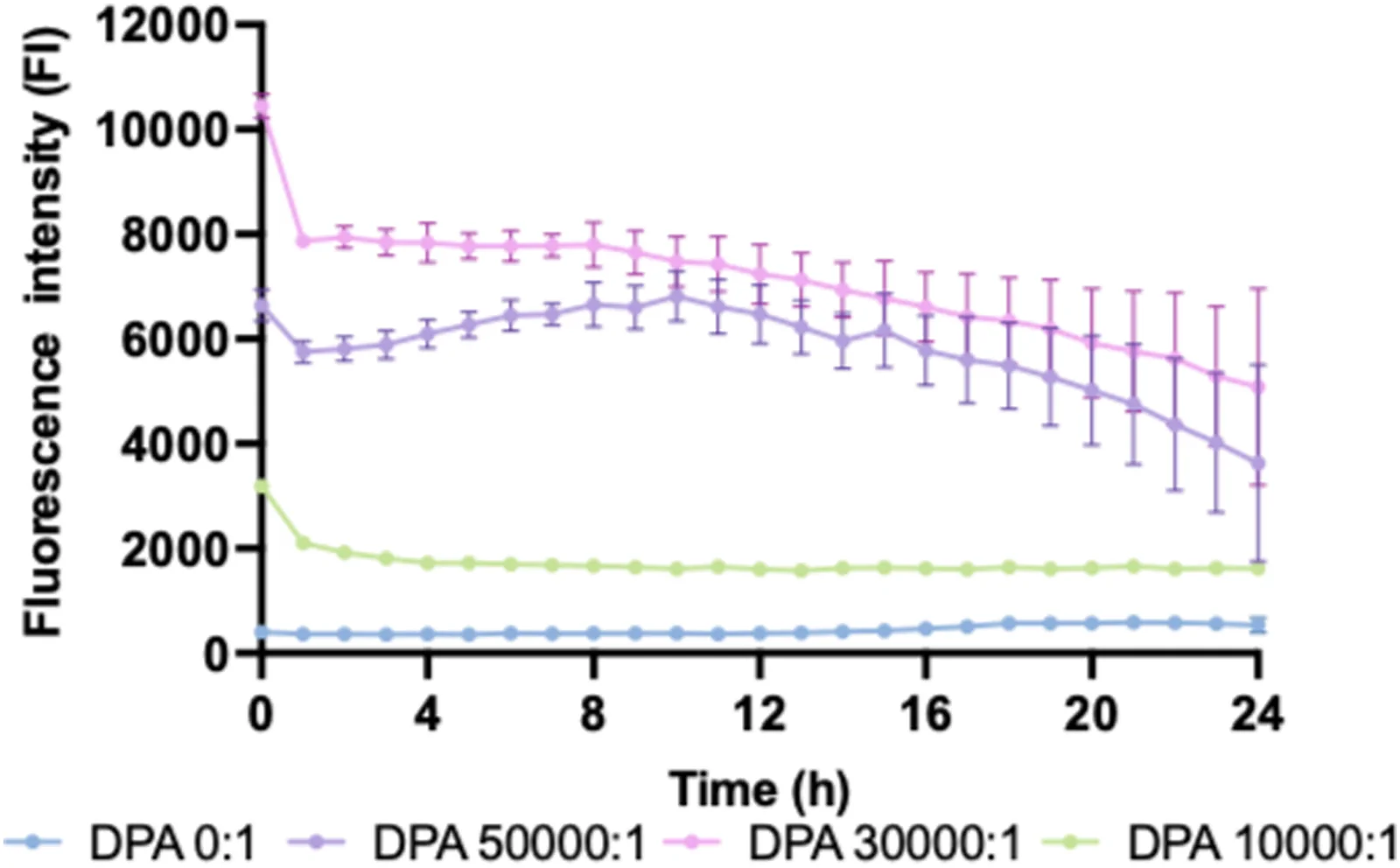
**FRET-based stability analysis of sEVs modified with DPA-Cy3**. Fluorescence intensity over 24 h at 37 °C for different molecules DPA:EV ratios, after subtraction of the phospholipid-only control. Data are presented as mean ± SD (n = 3). Fluorescence scans for the set-up of the method on Fig. S3.

Then, FRET was used to assess the temporal stability of DPA-based surface modification in eGFP-labeled sEVs. By tracking eGFP quenching alongside Cy3 emission over time, the assay reports whether DPA–Cy3 remains stably inserted in the vesicle membrane or whether a loss of FRET signal occurs, consistent with lipid desorption, membrane rearrangement, or aggregate-related instability. In Fig. 4, fluorescence kinetics are shown after subtracting the matched phospholipid-only controls (DPA–Cy3 without vesicles) for each concentration, providing a non-destructive, time-resolved readout of labeling stability. At higher lipid:EV ratios (50,000:1 and 30,000:1), the FRET signal was initially stable during the first ∼8 h at 37 °C but then progressively declined, suggesting a gradual redistribution and/or partial release of DPA–Cy3 from the vesicle surface over time. This behavior is consistent with excessive PEG-lipid packing, which can promote steric crowding and local phase separation in the membrane, ultimately reducing effective FRET coupling and apparent stability. In contrast, the 10,000:1 condition produced a lower absolute signal (as expected from fewer DPA molecules per vesicle) yet remained essentially stable across 24 h, and consistently above the negative control, indicating sustained incorporation of DPA–Cy3 and a more homogeneous, stable vesicle population. Overall, the FRET data confirm that DPA–Cy3 remains associated with sEVs and highlight that insertion density is a key determinant of stability: higher ratios yield stronger but less durable signals, whereas moderate loading provides more stable fluorescence and vesicle integrity over time.

To note, that given the comparative and formulation-oriented focus of this work, vesicles were characterized using complementary physicochemical and single-particle approaches, while broader EV characterization analyses recommended by MISEV2023 [25] were beyond the scope of the present study.

### 3.4 Cellular uptake of the modified sEVs in 2D cultures

Since the ultimate goal of engineering sEVs is their use as delivery vehicles, we assessed whether surface-modified vesicles remain able to interact with and be internalized by recipient cells. Uptake was primarily quantified using membrane-associated fluorescence, combining a general membrane dye (ACO600) with our DPA-based modification tracked through fluorescent DPA labeling. This dual readout provides a robust measure of vesicle–cell interaction and internalization while ensuring that the detected signal corresponds to membrane-associated labeling on engineered sEVs.

**Fig. 5:**
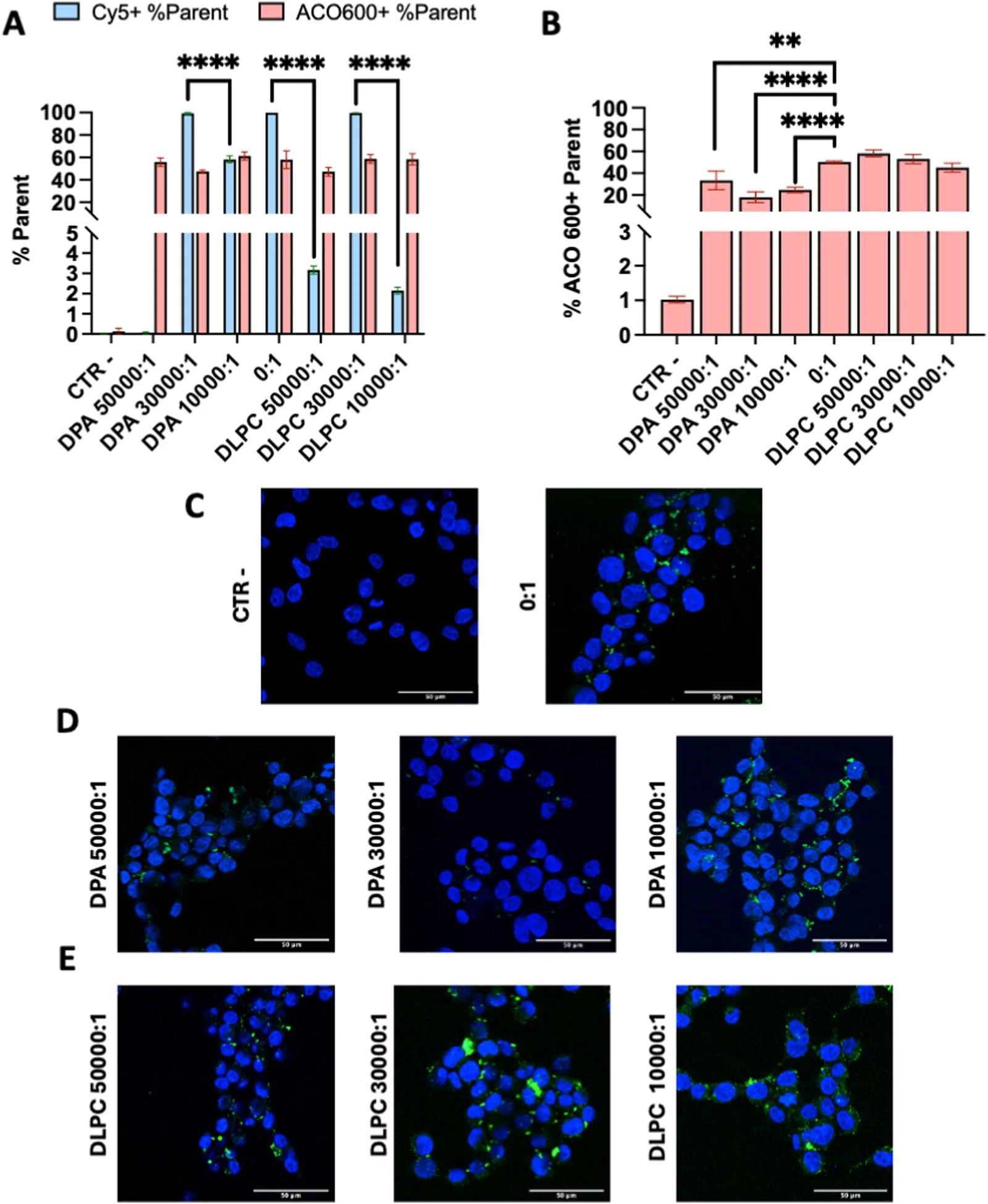
**Analysis of sEVs cellular uptake.** (**A**) Uptake in parental HEK293 cells quantified with ACO600 dye and DPA-Cy5; and (**B**) Uptake in BEAS-2B pulmonary epithelial cells quantified by ACO600; both by flow cytometry. Example confocal micrographs of HEK293 cells: (**C**) Controls, (**D**) DPA-modified sEVS and (**E**) DLPC-modified sEVs. DAPI (nuclei, blue), eGFP (tetraspanins CD81, green). Merge images, for full channels details see Fig. S5. Statistical analysis: ns = not significant, ****p < 0.0001, ***p < 0.001, **p < 0.01 and *p < 0.5.

Using ACO600-labeled sEVs, we observed substantial uptake in parental HEK293 cells (Fig. 5A.), consistent with the expected preference of EVs for their cell type of origin. Uptake was broadly comparable across conditions, with only modest decreases for selected formulations, suggesting that a denser lipid coating can partially reduce vesicle–cell interactions, likely by masking membrane components involved in binding and internalization. Fluorescent DPA labeling further confirmed that cellular signal was associated with DPA-modified vesicles, and lipid-only controls were included to exclude contributions from free lipid aggregates (Fig. 5A). In BEAS-2B bronchial epithelial cells (Fig. 5B), overall uptake was lower than in HEK293, consistent with reduced interaction in a heterologous cell type, and the reduction was more evident for DPA-containing formulations, in line with a steric “shielding” effect of PEGylated lipids. Uptake was also monitored via the eGFP signal from the CD81 fusion protein (Fig. S4), as established in our previous work (publication submitted), but this readout is typically less sensitive in conventional flow cytometry because the fluorescence per vesicle is lower and can be further attenuated after internalization (e.g., in intracellular compartments). For this reason, membrane dyes and DPA-based labeling provided a more reliable quantification of sEVs uptake.

To complement flow cytometry, uptake was evaluated by confocal microscopy using eGFP visualization. As shown in Fig. 5C–E, eGFP fluorescence appeared as discrete intracellular puncta across all conditions, confirming that engineered sEVs retain the capacity for cell interaction and internalization. Confocal microscopy can reveal vesicle clusters as fluorescent dots by integrating weak signals over space and time; while it is not suited for absolute quantification, it provides qualitative information on intracellular localization and relative differences between conditions. Signal intensity varied with lipid ratio, appearing strongest at intermediate conditions (notably DPA 10,000:1 and DLPC 30,000:1) that previously showed stable incorporation without major aggregation, whereas higher lipid ratios (e.g. 50,000:1) tended to show weaker fluorescence, consistent with partial uptake reduction due to excessive surface coating (and, for DPA, PEG-related shielding). Overall, these data indicate that surface-modified vesicles remain uptake-competent and suggest that intermediate lipid ratios best balance stability with cellular interaction.

### 3.5 Evaluation of surface-engineered sEVs interaction with mucus barriers

The next step was to evaluate how DPA and DLPC surface modifications affect the ability of sEVs to traverse the airway mucus barrier, the first physiological obstacle encountered before reaching the pulmonary epithelium. Experiments were restricted to lipid ratios previously identified as functional and aggregation-free, to focus on formulations with translational potential. Mucus permeability was assessed using a reconstituted mucin gel (10 mg/mL) in a Transwell® setup that provides a controlled, reproducible mucus-only barrier, previously validated by *Sarmento's* group [54]. Vesicles were applied to the apical side and transport was quantified over 24 h using cumulative permeability and P_app_, with vesicle amounts derived from a fluorescence-particle calibration curve (Fig. S6). Although this simplified model does not include epithelial complexity, it isolates mucus as the key barrier to determine how surface engineering modulates sEV mobility. This reductionist setup is therefore intended to model the initial mucus-diffusion step independently of subsequent epithelial and endothelial transport processes. Selected ratios were chosen based on the previous physicochemical screening, prioritizing stable formulations with clear evidence of lipid incorporation. For DPA, 10,000:1 and 30,000:1 were tested, while 50,000:1 was excluded because its smaller apparent diameter suggested possible PEG crowding or excess-lipid effects. For DLPC, 30,000:1 and 50,000:1 were selected because they showed the clearest size increase, whereas 10,000:1 showed a weaker modification profile and was not prioritized for biological evaluation. Unmodified sEVs were used as the relevant internal reference to assess the relative impact of each surface modification on vesicle transport across the mucin barrier.

**Fig. 6:**
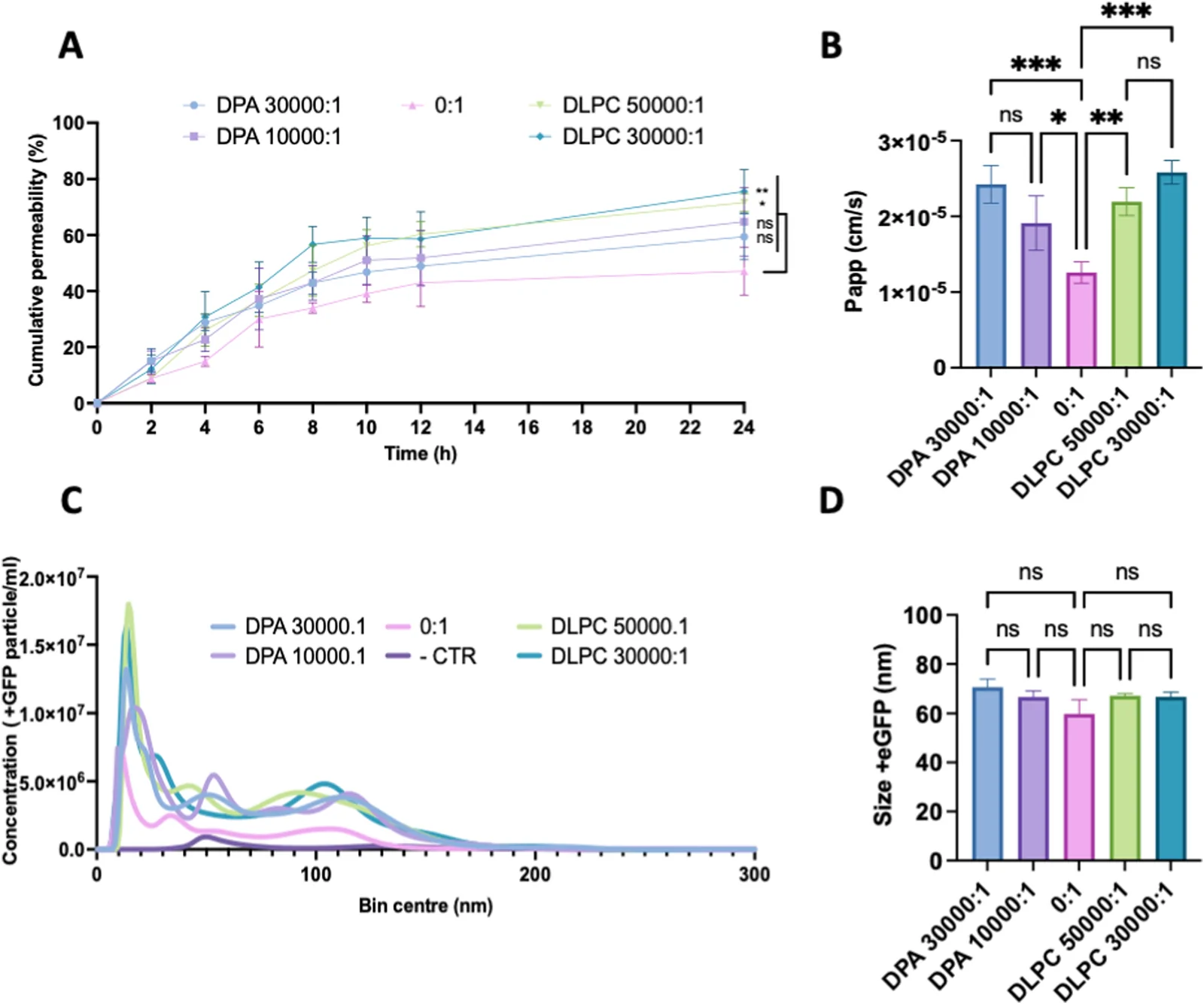
**Permeability studies of surface-modified EVs through a mucin barrier (10 mg/mL).** (**A**) Cumulative permeability (%) over time for EVs with different surface modifications: DPA (30,000:1 and 10,000:1), DLPC (50,000:1 and 30,000:1), and unmodified control (0:1). (**B**) Apparent permeability coefficient (P_app_) values calculated at the 4 h. **(C**) Concentration-size distribution of sEVs in the basolateral compartment after 24 h. DPA (30,000:1 and 10,000:1), DLPC (50,000:1 and 30,000:1), unmodified control (0:1) and buffer control (-CTR), as measured by NTA. (**D**) Average size of recovered + eGFP vesicles, as NTA measurements. Data are presented as mean ± SD (n = 3). Statistical significance was determined by one-way ANOVA followed by Sidak's (A, B), for selected comparisons; or Turkey's (C, D), for multiple-comparison of all pairwise comparisons test: *p < 0.05, **p < 0.01, ***p < 0.001, ns = not significant.

As shown in Fig. 6A., unmodified sEVs (0:1) displayed the lowest cumulative permeability, plateauing at ∼40%, indicating limited diffusion through the mucin layer. DPA-modified vesicles (30,000:1 and 10,000:1) showed only a modest increase (to ∼60%) that was not statistically significant. In contrast, DLPC-modified sEVs-particularly at 30,000:1-exhibited a clear improvement, reaching ∼80% cumulative permeability. Fig. 6B reports P_app_ calculated at 4 h during the quasi-linear transport phase to avoid early lag and late saturation effects. Control sEVs again showed the lowest P_app_. DPA increased P_app_ relative to control, with DPA 30,000:1 yielding the highest rate among DPA conditions, although differences between DPA ratios were not significant (Fig. 6B.). DLPC performed best overall, with DLPC 30,000:1 producing the largest (and statistically significant) increase in permeability; DLPC 50,000:1 also improved transport but without clear separation between DLPC doses. Overall, both lipid modifications enhanced mucus diffusion, with 30,000:1 emerging as the most favorable ratio for each lipid. The limited separation in 24 h cumulative permeability among DPA conditions likely reflects time-course convergence as profiles approach saturation: PEGylation can reduce mucoadhesive interactions and accelerate early diffusion (higher P_app_), but this advantage may not translate into substantially higher cumulative transport over extended periods [18]. By contrast, DLPC modification supported more sustained transport, resulting in higher cumulative permeability before saturation.

To confirm that the permeability signal reflected intact vesicles rather than free dye, protein or degraded fragments, basolateral samples were analyzed by NTA. In addition with the fluorescence readouts, the control (0:1) showed lower particle concentrations across the EV size range, whereas both DPA- and DLPC-modified samples displayed higher particle counts (Fig. 6C.), indicating that more vesicles reached the receiver compartment. DLPC samples tended to show the highest concentrations, in line with their superior permeability. Mean particle size remained comparable across conditions (Fig. 6D.; ns), ruling out size-dependent bias, aggregation, or fragmentation as drivers of the observed differences. Together, these results support that lipid engineering, especially DLPC, enhances the transport of intact sEVs across a mucin barrier while preserving vesicle size and integrity. The improved performance of DLPC-modified sEVs may be attributed to the zwitterionic phosphocholine headgroup and short acyl chains of DLPC, which can promote a hydrated, near-neutral, and more fluid vesicle surface with reduced adhesive interactions with mucin. In contrast, although DPA may decrease nonspecific interactions through PEGylation, the bulky PEG(5000) chains can increase steric hindrance and potentially limit efficient diffusion through the mucus mesh. These observations are consistent with previous studies showing that EV surface engineering with hydrophilic or PEG-based motifs can improve transport across mucus barriers by reducing adhesive interactions with mucins [35,73]. In particular, PEGylated milk EVs were previously reported to exhibit enhanced mucus penetrability and improved siRNA delivery in an oral model [35], while a more recent study showed that hydrophilic and zwitterionic surface motifs can further enhance EV permeability through mucus while preserving functional delivery [73]. In this context, our results extend these concepts to the pulmonary setting and indicate that DLPC at 30,000:1 provides the most favorable balance among the formulations tested, outperforming DPA-modified vesicles in cumulative permeability and diffusive behavior in a lung-relevant mucin model. Thus, although no direct membrane biophysical or mucin-binding measurements were performed in the present study, the observed formulation-dependent transport behavior is consistent with previous reports showing that hydrophilic and zwitterionic surface motifs can reduce adhesive interactions with mucus and improve vesicle diffusion across mucosal barriers [35,58,73,74]. In parallel, PEG-lipid insertion has been reported to reduce non-specific interactions through steric shielding, but this same effect may also attenuate productive interactions with recipient cells depending on the biological context [35,58,73,74].

To further probe how surface engineering alters sEV mobility within mucus, MPT was performed using the same vesicle formulations and concentrations as in the permeability assays. Vesicles were incubated for 1 h in a reconstituted mucin gel (10 mg/mL) to allow equilibration, and trajectories were recorded by fluorescence microscopy using the intrinsic eGFP signal, enabling single-particle quantification of displacement over time.

**Fig. 7:**
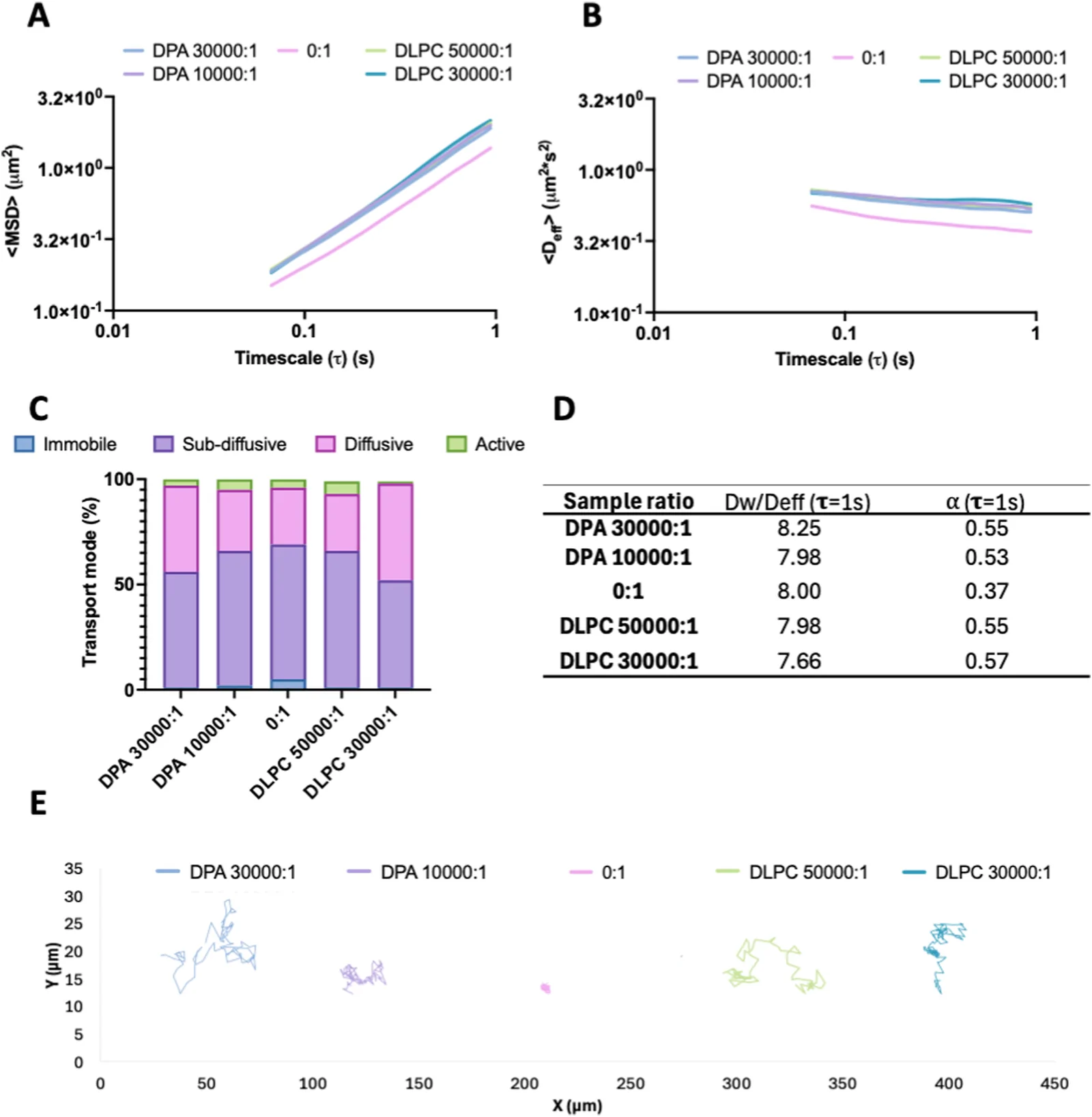
**Multiple Particle Tracking (MPT) analysis of EV mobility in a 10 mg/mL mucin solution.** (**A**) Mean Square Displacement (MSD) plotted as a function of timescale. (**B**) Effective diffusion coefficient (D_eff_) calculated from particle trajectories. (**C**) Classification of transport modes based on trajectory analysis: immobile, sub-diffusive, diffusive, and active. MPTHub software were used to obtain final values. (**D**) Diffusion parameters of surface-modified sEVs in mucin. (**E**) Representative trajectories of a single model surface-modified sEV in a 10 mg/mL mucin gel. Individual particle displacements are shown for DPA 30,000:1, DPA 10,000:1, unmodified control (0:1), DLPC 50,000:1, and DLPC 30,000:1 sEVs over the same time window.

MPT showed that mean squared displacement (MSD) increased with time in all conditions (Fig. 7A.), but unmodified sEVs (0:1) consistently displayed the lowest MSD, indicating stronger confinement within the mucin network. Both DPA- and DLPC-modified vesicles exhibited higher MSD values, reflecting improved mobility, although differences between lipid types were modest. MSD slopes (α) were <1 for all samples, consistent with sub-diffusive transport in viscoelastic mucus. Effective diffusion coefficients (D_eff_), derived from MSD (Fig. 7B.), followed the same trend: controls had the lowest D_eff_, whereas surface-modified vesicles diffused faster. D_eff_ decreased with increasing timescale, consistent with a transition from short-range motion to progressively hindered transport as vesicles encounter steric constraints in the mucin mesh.

Analysis of transport modes (Fig. 7C) highlighted a shift in behavior upon modification: control vesicles showed the highest fraction of sub-diffusive trajectories, while DPA 30,000:1 and DLPC 30,000:1 reduced the sub-diffusive fraction and increased the proportion of trajectories classified as diffusive, indicating a more permissive mobility pattern. Quantitatively (Fig. 7D), all formulations experienced a similar overall hindrance in mucus (D_w_/D_eff_ at τ = 1 s ≈ 7.7–8.3), but the anomalous exponent α increased with lipid modification (from 0.37 for control to ∼0.53–0.57 for modified vesicles), consistent with partial release from mucin constraints and motion closer to normal diffusion.

Representative trajectories (Fig. 7E) visually reinforced these findings: unmodified sEVs showed short, confined paths, whereas DPA- and DLPC-modified vesicles displayed longer, more continuous displacements, with DPA 30,000:1 and DLPC 30,000:1 showing the most extended trajectories. Overall, MPT confirms that lipid functionalization increases the fraction of mobile vesicles in mucus and shifts their transport toward less confined, more diffusive behavior.

### 3.6 Evaluation of surface-engineered sEVs capacity to cross a 3D pulmonary barrier model

To complement the mucus-only permeability assays, the same sEV formulations were evaluated in a more physiologically relevant 3D pulmonary barrier co-culture on Transwell® inserts, with NCI-H441 epithelial cells on the apical side and HPMEC-ST1.6R endothelial cells on the basolateral side. This epithelial–endothelial arrangement recapitulates key features of the distal lung/alveolar-capillary interface, including polarization, tight-junction formation, and selective transport). Importantly, this model was not intended to reproduce a fully mucus-rich conducting airway epithelium. While NCI-H441 cells are not strongly mucus-producing, they release low levels of mucins and surfactant that support a relevant ALI microenvironment. The model has been described and validated in previous studies and provides a robust compromise between physiological relevance, reproducibility, and throughput for screening EV/nanoparticle translocation across combined mucus and cellular barriers [52,54].

**Fig. 8:**
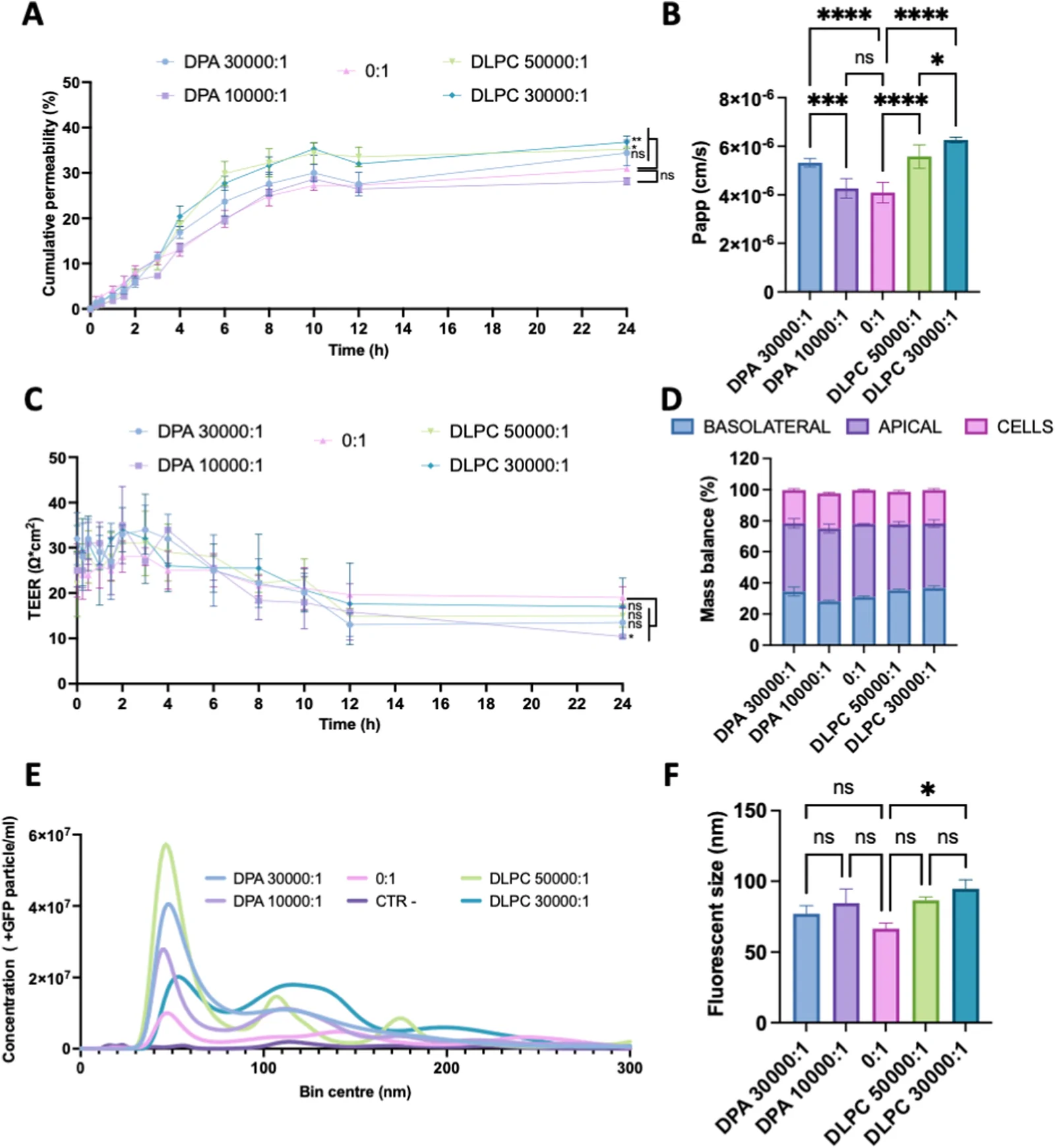
***Permeability of surface-modified sEVs across the 3D pulmonary cell barrier.*** (**A**) Cumulative permeability (%) determined from fluorescence in the basolateral compartment over 24 h. (**B**) P_app_ calculated at 4 h. (**C**) TEER values measured during the assay. (**D**) Mass balance at 24 h. **(E**) Concentration-size distribution of sEVs in the basolateral compartment after 24 h. DPA (30,000:1 and 10,000:1), DLPC (50,000:1 and 30,000:1), unmodified control (0:1) and buffer control (-CTR), by fluorescent NTA. (**F**) Average size of recovered + eGFP vesicles, by NTA. Data are presented as mean ± SD (n = 3). Statistical analysis was performed using one-way ANOVA with followed by Sidak's multiple comparison test (A to D) or Tukey's multiple-comparison test for all pairwise comparisons (E, F): *p < 0.05, **p < 0.01, ***p < 0.001, ****p < 0.001, ns = not significant.

After a preliminary compatibility check confirmed no loss of viability under the tested exposure conditions (Fig. S7) and TEER monitoring verified appropriate barrier formation and integrity prior to transport experiments (Fig. S8), cumulative permeability was quantified from basolateral fluorescence over 24 h (Fig. 8A). All conditions showed a rise until ∼10 h followed by a plateau, consistent with saturation of transport pathways and/or depletion of readily translocatable vesicles. Unmodified sEVs (0:1) exhibited the lowest permeability throughout. DPA-modified vesicles (30,000:1 and 10,000:1) largely overlapped the control, indicating no measurable gain in overall passage, consistent with PEG-driven steric shielding that can reduce vesicle–cell interactions in a cellular barrier. In contrast, DLPC modification clearly enhanced translocation: DLPC 30,000:1 and 50,000:1 reached ∼35% cumulative permeability after 24 h (with 30,000:1 trending slightly higher), supporting that DLPC incorporation promotes more efficient transport across the epithelial–endothelial cell interface, likely by favoring vesicle–cell interaction and transcellular trafficking. P_app_ values were calculated at 4 h (linear phase; Fig. 8B), again showing the lowest transport rate for unmodified vesicles and the highest values for DLPC 30,000:1 and 50,000:1, consistent with faster early translocation than both control and DPA conditions. During the assay, TEER decreased from ∼25 to 30 to ∼20 Ω cm^2^ across all groups (Fig. 8C), indicating modest barrier relaxation over 24 h that was comparable between conditions and therefore more consistent with a general assay effect than with formulation-specific toxicity. Mass balance analysis (Fig. 8D) showed substantial apical retention (∼45%) and a sizeable cell-associated fraction (∼20%), supporting that the barrier and intracellular trafficking are rate-limiting steps for basolateral recovery within the assay window, in line with the superior basolateral transport observed for DLPC-engineered vesicles.

To verify that basolateral fluorescence reflected transported intact sEVs rather than free signal, basolateral samples were analyzed by fluorescence-NTA, which enables particle counting and size profiling while specifically detecting engineered fluorescent vesicles. In agreement with permeability measurements, unmodified sEVs yielded the lowest recovered fluorescent particle counts, whereas DPA 30,000:1 and DLPC 30,000:1 showed the highest basolateral vesicle concentrations (Fig. 8E), and the buffer control showed no fluorescent particles. Recovered vesicles remained within the expected ∼50–100 nm size range with only minor shifts (Fig. 8F), supporting structural preservation after crossing. Overall, these data indicate that surface engineering can increase the number of intact sEVs translocating across a 3D pulmonary barrier, with DLPC providing the most consistent improvement in transport kinetics and overall passage, particularly at intermediate lipid loading. This tendency toward apical retention may reflect the steric shielding associated with PEG-lipid insertion, which has previously been shown to reduce EV-cell interactions, rather than a complete loss of vesicle uptake capacity. This distinction between DPA and DLPC becomes particularly relevant when viewed in the context of previous EV-engineering studies. PEGylated EV formulations have been reported to improve colloidal behavior and reduce non-specific interactions [74], but this same shielding effect may also attenuate productive cell interactions depending on the biological barrier considered. Our data are in line with this concept: DPA-functionalized EVs displayed only moderate barrier transport and a tendency toward apical retention, whereas DLPC modification better preserved the balance between reduced mucus-associated hindrance and productive epithelial translocation. Importantly, the present study adds a lung-specific perspective by demonstrating this trade-off across sequential pulmonary barriers, including both mucus and an epithelial-endothelial co-culture interface.

To clarify whether surface-modified sEVs traverse the pulmonary co-culture mainly through uptake-associated transcellular transport, and to identify formulations prone to intracellular retention rather than efficient basolateral release, uptake was examined using complementary imaging and flow-based approaches. The aim was to connect permeability outcomes with vesicle–cell interactions by determining where vesicles accumulate after internalization (apical epithelial layers versus deeper regions approaching the endothelial side) while recognizing that the present setup does not fully exclude a contribution from paracellular passage.

**Fig. 9:**
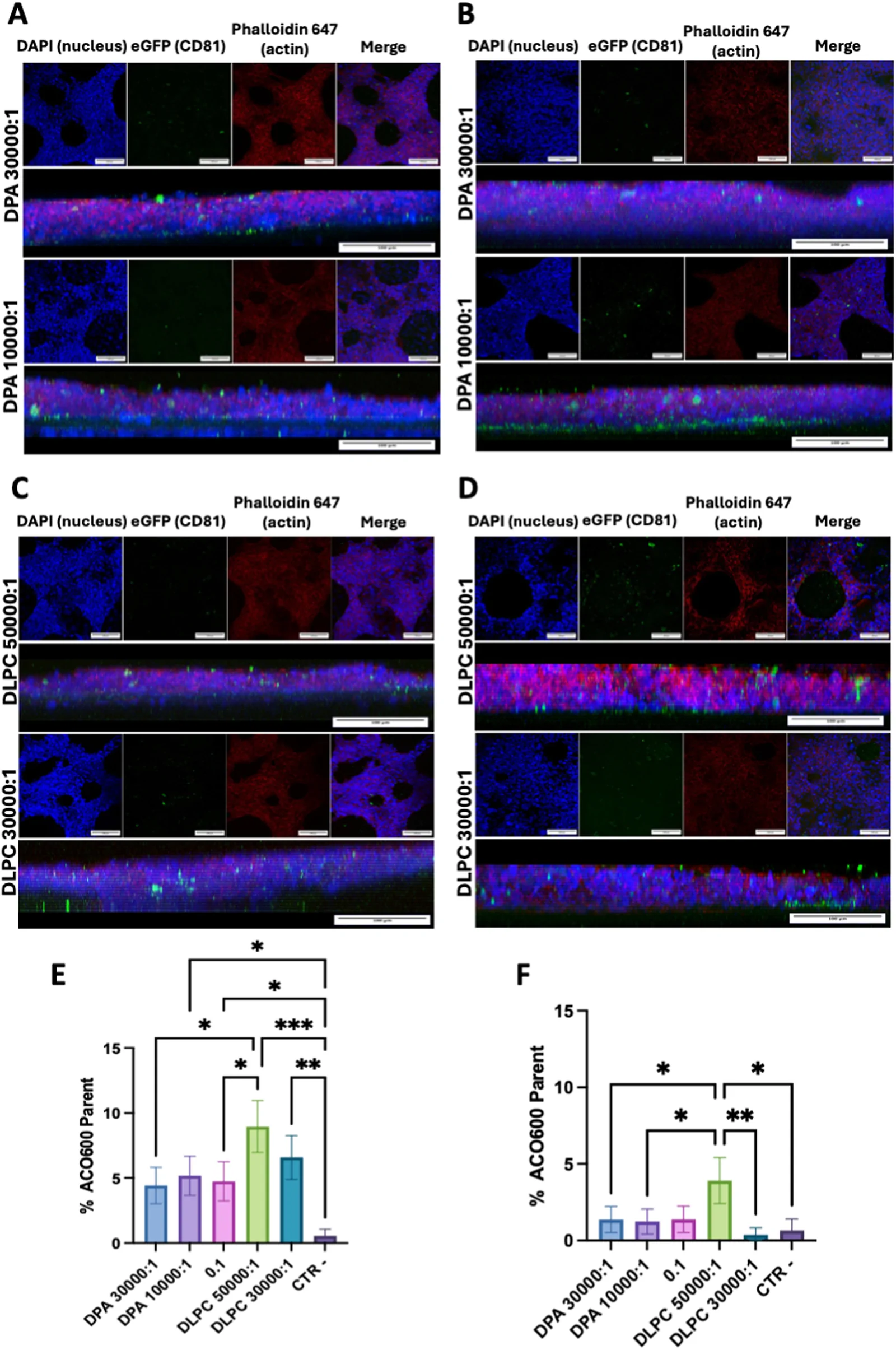
Analysis of surface-modified sEVs uptake in the 3D pulmonary barrier model. NCI-H441 epithelial cells (apical side) and HPMEC-ST1.6R endothelial cells (basolateral side) were cultured on Transwell® inserts and incubated with eGFP-labeled sEVs. *(****A-D****)****Confocal microscopy****: (****A****) DPA-modified sEVs, 8 h. (****B****) DPA-modified sEVs, 16 h. (****C****) DLPC-modified sEVs, 8 h. (****E****) DLPC-modified sEVs, 16 h. Nuclei were stained with DAPI (blue), actin filaments with phalloidin 647 (red), and vesicles detected by eGFP (green).* Images were acquired using a 40× objective*. Scale bars:* 100 μm *all images. (****E-F****)****Flow cytometry of ACO600-labeled sEVs****(****E****) apical epithelial cells (NCI-H441) and (****F***) basolateral endothelial cells (HPMEC-ST1.6R) after 8 h of incubation with unmodified and surface-modified vesicles. Data are presented as mean ± SD (n = 3). Statistical significance was determined by one-way ANOVA followed by Sidak's multiple comparisons test: *p < 0.05, **p < 0.01, ***p < 0.001.

Confocal microscopy of fixed Transwell® inserts confirmed that the co-culture could be reliably visualized (Fig. S10), with NCI-H441 epithelial layers on the apical side and HPMEC-ST1.6R endothelial cells on the basolateral side separated by the porous membrane (often visible as a thin line). Minor irregularities, likely introduced during post-fixation processing, did not compromise overall barrier integrity, consistent with TEER validation prior to the assay. Imaging at 8 h and 16 h captured an early phase aligned with the transport peak observed in permeability studies and a later, conventional uptake endpoint. As expected, the negative control showed no fluorescent puncta at either time point, confirming signal specificity (Fig. S10). Unmodified sEVs (0:1; Fig. S11) produced detectable puncta but these were largely confined to the apical epithelial surface, matching their limited penetration and lower permeability. DPA-modified vesicles showed intermediate behavior: puncta were observed within mid-layers at 8 h, suggesting that PEGylation can facilitate penetration beyond the apical surface, but by 16 h DPA 10,000:1 displayed more evident apical retention—consistent with aggregation and reduced onward progression—whereas DPA 30,000:1 showed fewer apically retained puncta, aligning with its comparatively better transport profile (Fig. 9A and B). In contrast, DLPC-modified vesicles exhibited the most favorable distribution: puncta were already present within intermediate layers at 8 h and extended to deeper regions by 16 h, consistent with more effective progression through the barrier. Notably, DLPC 30,000:1 appeared to show less epithelial retention than DLPC 50,000:1, in agreement with its superior permeability performance (Fig. 9C and D). Co-staining with DAPI (nuclei) and phalloidin (actin) supported that puncta were predominantly intracellular (cytoplasmic) rather than merely surface-bound, with superficial signal mainly in localized regions of discontinuity.

To quantify uptake independently in apical and basolateral compartments, flow cytometry was performed at 8 h using ACO600 membrane labeling to ensure robust detection regardless of the variable eGFP readout. In the apical epithelial population (Fig. 9E), the negative control remained near baseline, confirming that fluorescence reflected vesicle-associated signal, but the fraction of positive cells stayed below ∼15% across groups, likely reflecting a low vesicle-to-cell ratio in this dense co-culture. Uptake of unmodified sEVs was similar to both DPA conditions, indicating that PEGylation did not measurably enhance early apical internalization, whereas DLPC increased the fraction of ACO600-positive epithelial cells, most clearly at DLPC 50,000:1. In the basolateral endothelial population (Fig. 9F), only DLPC 50,000:1 showed a significant fluorescence increase over the negative control (still <5% positive cells), suggesting that higher DLPC incorporation promotes progression across the epithelial layer and delivery to endothelial cells, consistent with an early transcellular transport component. Overall, these data support formulation-dependent intracellular handling: DPA formulations show more evidence of apical retention and/or limited detectable uptake (potentially influenced by aggregation and altered endocytic processing), while DLPC, particularly at higher loading, enhances epithelial uptake and early basolateral transfer. The low positive fractions in both compartments also indicate that future studies using higher vesicle doses and additional time points would better resolve uptake and translocation kinetics in this model.

**Fig. 10:**
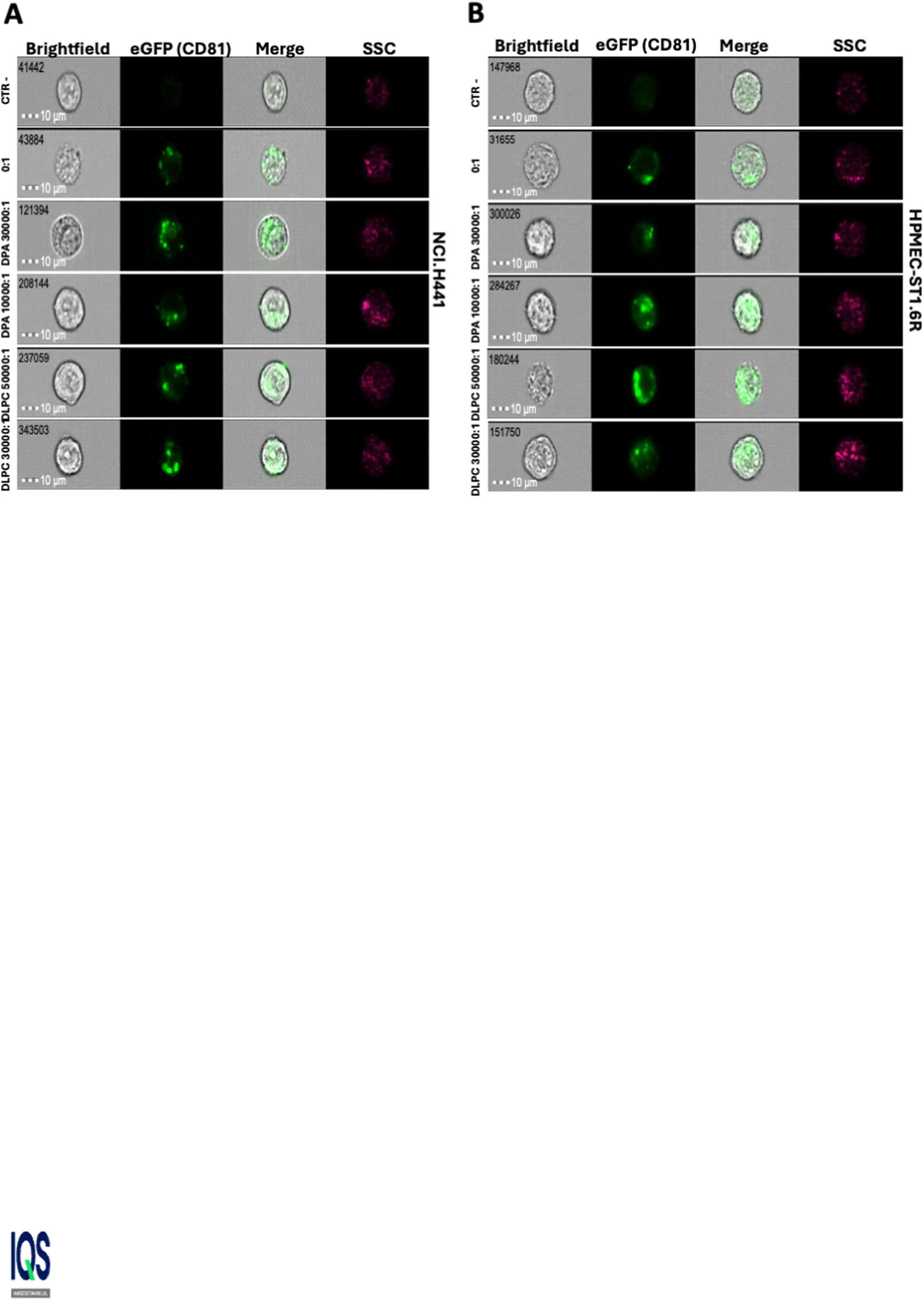
**Representative images from Imaging Flow Cytometry (ImageStream) showing uptake of surface-modified sEVs in the 3D pulmonary barrier model.** (**A**) Apical epithelial cells (NCI-H441) and (**B**) basolateral endothelial cells (HPMEC-ST1.6R) after 8 h of incubation with unmodified (0:1) or surface-modified vesicles (DPA 30,000:1, DPA 10,000:1, DLPC 50,000:1, DLPC 30,000:1). Channels shown: brightfield, eGFP signal (vesicles/CD81), merged image, and side scatter (SSC). Each row corresponds to a representative single cell from a dataset of >60,000 analyzed per condition. Scale bar = 10 μm.

**Fig. 11:**
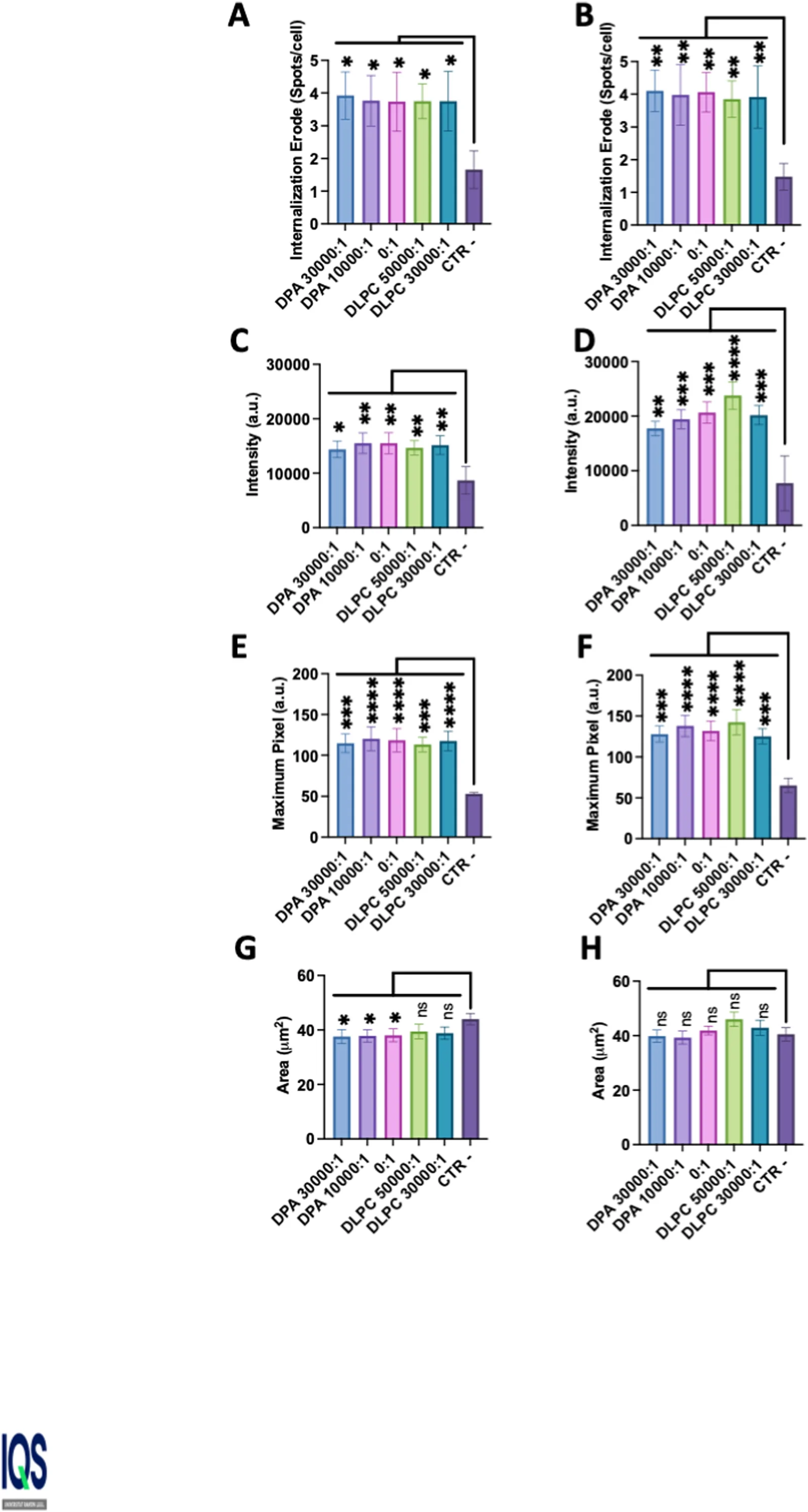
**Quantitative ImageStream analysis of surface-modified sEVs uptake in the 3D pulmonary barrier model.** (**A**) Internalization Erode in apical cells (NCI-H441). (**B**) Internalization Erode in basolateral cells (HPMEC-ST1.6R). (**C**) Fluorescence intensity per cell in apical cells. (**D**) Fluorescence intensity per cell in basolateral cells. (**E**) Maximum Pixel in apical cells. (**F**) Maximum Pixel in basolateral cells. (**G**) Cell area of apical cells. (**H**) Cell area of basolateral cells. Data are presented as mean ± SD (n = 3). Statistical significance was determined by one-way ANOVA followed by Sidak's multiple comparisons test: *p < 0.05, **p < 0.01, ***p < 0.001, ****p < 0.0001, ns = not significant.

To corroborate the confocal and flow-cytometry uptake data, imaging flow cytometry (ImageStream) was used, combining single-cell imaging with quantitative analysis (≥60,000 cells/condition) to distinguish true intracellular signal from surface-bound vesicles in both apical (NCI-H441) and basolateral (HPMEC-ST1.6R) cell populations. In apical cells (Fig. 10A.), the negative control showed no eGFP signal, unmodified sEVs yielded only weak/occasional peripheral fluorescence, and both DPA- and DLPC-modified vesicles displayed clearer punctate cytoplasmic signal, with DLPC often showing more perinuclear localization consistent with endosomal trafficking. In basolateral cells (Fig. 10B.), fluorescence remained low across conditions (Fig. 11.), indicating that only a small fraction of vesicles reached endothelial cells within the analyzed time window. Quantitatively, internalization metrics confirmed genuine uptake for all fluorescent sEV conditions versus the negative control, without major differences between unmodified and surface-modified vesicles in either compartment. Total fluorescence intensity was similar across vesicle conditions in apical cells, whereas in basolateral cells, DLPC 50,000:1 showed significantly higher intensity than the unmodified control, aligning with earlier flow cytometry and supporting enhanced endothelial delivery/retention for this formulation. Intracellular signals of accumulation (e.g., endosomal) were higher than the negative control but not meaningfully different between formulations, and cell area was unchanged across groups, indicating no detectable morphological changes. Overall, ImageStream provided multiparametric confirmation that vesicles are internalized in apical cells under all conditions, while DLPC 50,000:1 uniquely increases vesicle-associated signal in the endothelial layer, consistent with enhanced transcellular-associated transport, although the relative contribution of paracellular passage cannot be fully resolved in the present setup.

In summary although the combined imaging, cell-associated fluorescence, and basolateral recovery data support a relevant contribution of uptake-associated transcellular transport, the present experimental setup does not fully exclude a contribution from transient paracellular passage.

## 4 Conclusions

In this study, we demonstrate that post-secretory surface engineering of sEVs with exogenous phospholipids is an effective strategy to modulate their behavior across lung-relevant barriers 3D models without compromising vesicle integrity or cell viability. Incorporation of DLPC or DPA into sEV membranes enabled fine-tuning of surface charge and colloidal properties while maintaining nanoscale size and structural stability at optimized lipid-to-vesicle ratios. Notably, excessive DPA embedding led to aggregation, underscoring the importance of carefully controlling lipid density during formulation. Across a reconstituted mucin gel as well as in a 3D pulmonary epithelial-endothelial co-culture, both lipid-engineered sEVs showed superior transport compared with unmodified vesicles, which were largely trapped within the mucus network. However, while DPA-modified vesicles displayed moderate but significant permeability, with a tendency toward retention at the apical epithelium, consistent with PEG-mediated reduction of non-specific interactions rather than promotion of deep translocation; DLPC at a 30,000:1 lipid-to-vesicle ratio achieving the highest cumulative permeability, sustained P_app_ values, and more diffusive motion profiles. Importantly, vesicles detected in the basolateral compartment remained structurally intact, confirming that the enhanced signal reflected true vesicle transport rather than dye leakage or vesicle disruption. Neither DLPC nor DPA induced measurable alterations in cell morphology, barrier integrity, or viability, confirming their biocompatibility in this advanced lung model. Overall, our data identify DLPC at a 30,000:1 ratio as the formulation that best balances mucus penetration, epithelial permeability, and efficient barrier crossing associated with improved epithelial interaction and basolateral recovery, positioning it as a promising candidate for pulmonary delivery applications in which vesicles must cross the full lung barrier, for example to reach the vasculature or deeper tissues. This strategy could be particularly relevant for diseases requiring efficient delivery across mucus and epithelial barriers, including chronic inflammatory lung diseases such as cystic fibrosis, asthma, and chronic obstructive pulmonary disease, as well as pulmonary fibrosis, lung cancer, and systemic conditions where inhaled vesicles could serve as a non-invasive route to reach the bloodstream. In contrast, DPA appears more suitable for applications prioritizing epithelial engagement and colloidal stability, where extensive translocation is not required. These findings highlight the potential of rational phospholipid engineering to tailor sEVs performance according to specific therapeutic objectives and reinforce the value of 3D lung-mimetic models for dissecting how subtle changes in surface chemistry translate into distinct transport profiles and provide a basis for future *in vivo* pulmonary studies aimed at evaluating biodistribution, retention, and functional performance after administration.

## CRediT authorship contribution statement

**Maria José Sanchez:** Writing – original draft, Methodology, Investigation, Conceptualization. **Pablo Leivar:** Writing – review & editing, Formal analysis. **Soraia Pinto:** Writing – review & editing, Methodology. **Helena Almeida:** Writing – review & editing, Methodology, Investigation. **Margalida Esmeralda Artigues:** Writing – review & editing, Formal analysis. **Bruno Sarmento:** Writing – review & editing, Validation, Supervision, Funding acquisition. **Martí Lecina:** Writing – review & editing, Supervision, Funding acquisition, Data curation, Conceptualization. **Cristina Fornaguera:** Writing – review & editing, Supervision, Funding acquisition, Formal analysis, Data curation, Conceptualization.

## Declaration of competing interests

The authors declare that they have no known competing financial interests or personal relationships that could have appeared to influence the work reported in this paper.

## Data Availability

Data will be made available on request.
