# Supplementary material for: Balancing stability and cellular interaction in surface-engineered small extracellular vesicles for pulmonary delivery
Source: Mater Today Bio. 2026 Sep 1;40:103637. doi: 10.1016/j.mtbio.2026.103637 (PMC13563590; doi:10.1016/j.mtbio.2026.103637)
Supplement: Supplementary file 1 [file mmc1.pdf]

Balancing stability and cellular interaction in surface-engineered small extracellular vesicles for pulmonary delivery

Maria José Sanchez[1], Pablo Leivar[2], Soraia Pinto[3], Helena Almeida[3], Margalida Esmeralda Artigues[4], Bruno Sarmento[3, 5], Martí Lecina*[1], Cristina Fornaguera*[1]

SUPLEMENTARY INFORMATION

Explanation S1. Expanded description of the method for the Quantification by LC-MS/MS (QTRAP®7500)

Explanation S2. *In vitro* cell uptake assays.

Explanation S3. QTRAP method for Lipid quantification.

Figure S1. Physicochemical characterization of +eGFP sEVs modified with different DPA:EV or DLPC:EV ratios by nanoparticle tracking analysis (NTA).

Figure S2. Representative plots from nano-flow cytometry analysis of sEVs after labeling with DPA-Cy5.

Figure S3: Fluorescence emission spectra used to determine optimal excitation/emission wavelengths for FRET measurements.

Figure S4: Flow cytometry analysis of cellular uptake of eGFP surface-modified sEVs.

Figure S5: Confocal microscopy analysis of sEVs uptake in HEK293 cells.

Figure S6: Linear correlation between fluorescence intensity (AU) and concentration of fluorescent (EVs/mL) and equation used for mucin permeability calculations.

Figure S7: Cytotoxicity of free phospholipids, unmodified sEVs, and surface-modified sEVs in pulmonary epithelial and endothelial cell.

Figure S8: Establishment and validation of the 3D pulmonary cell barrier model.

Figure S9: Linear correlation between fluorescence intensity (AU) and concentration of fluorescent (EVs/mL) and equation used for 3D pulmonary model permeability calculations.

Figure S10: Confocal microscopy analysis of a control 3D pulmonary barrier model.

Figure S11: Confocal microscopy analysis of non-modfied EVs incubated on a 3D pulmonary barrier model.

Explanation S1. Expanded description of the method for the Quantification by LC-MS/MS (QTRAP®7500)

The quantification of phospholipids (DPA and DLPC) incorporated onto the surface of sEVs was performed using a Triple Quad™ 7500 LC-MS/MS System-QTRAP® (Sciex). The surface modification reaction was carried out as described above. Prior to assembly, all reagents and sEVs suspensions were filtered through PTFE hydrophobic membranes, 0.22 µm pore size (Phenomenex, AF8-7702-12) to remove particulate matter and aggregates. Following the insertion/functionalization reaction, samples were purified by ultrafiltration using Amicon® centrifugal filters (as previously described), thereby separating the retentate fraction (containing modified sEVs) from the filtrate (wash fraction containing non-incorporated lipids). The recovered volume was adjusted to the initial reaction volume. Due to solubility and buffer compatibility issues, DPA-containing fractions were diluted in methanol, whereas DLPC-containing fractions were diluted in PBS. In both cases, dilutions were prepared to match the concentration range of the calibration curves employed for LC-MS/MS analysis. Samples were transferred into 0.5 mL glass vials (Phenomenex, AR0-3920-12) and 1 mL glass vials (Agilent, 8010-0542) prior to injection. Retentate and filtrate fractions were then processed according to the extraction and analytical protocol detailed below.

Chromatographic separation was achieved on a Kinetex C18 column, 1.7 µm, 100 Å (Phenomenex). The mobile phases were solvent A = ACN/H_2O (60:40, v/v) with 0.1% formic acid; solvent B = IPA/ACN (90:10, v/v) with 0.1% formic acid. For needle washing and reconstitution, solvent R3 = MeOH/IPA (1:1, v/v) and solvent R0 = ACN/IPA/H_2O (40:36:24, v/v/v) were employed. To clarify, in multiple reaction monitoring (MRM) mode, the first quadrupole (Q1) selectively filters the precursor ion, the second quadrupole (Q2) induces fragmentation in the collision cell, and the third quadrupole (Q3) isolates the characteristic product ion, allowing highly specific and sensitive detection of the target phospholipids. Quantification was carried out using positive electrospray ionization (ESI+). For DPA, the monitored transition was precursor ion *m/z* 749.1 to product ion *m/z* 735.0, while for DLPC the monitored transition was precursor ion *m/z* 622.90 to product ion *m/z* 184.00. Source parameters and collision energies (CE) were optimized for each transition using standards.

Calibration curves were generated independently for each analyte. DPA standards were prepared in methanol at concentrations ranging from 0.5 to 500 ppb, while DLPC standards were prepared in PBS over the same concentration range. Calibration curves were constructed by plotting the **absolute peak area** of the analyte versus the nominal concentration. Sample concentrations were interpolated from the calibration curve and normalized to the number of sEVs. Quality controls included matrix blanks, evaluation of carryover through wash runs, and confirmation of analyte identity by retention time and ion ratio relative to the standard. Data were processed using the Analyst® Software (Sciex), and results were expressed as phospholipid content per sEVs number, normalized to counts obtained by NTA following the last purification step. Data are reported as mean ± SD (n = 3). Signal stability was assessed through re-injected standards, and batches showing degradation or contamination were excluded.

Explanation S2. *In vitro* uptake assays detailed description.

Flow cytometry-based uptake efficiency was measured on a Beckman Coulter CytoFLEX S (Beckman Coulter, Germany). HEK293 and BEAS-2B cells were seeded in triplicate in 96-well plates (SPL Life Sciences, 30096) at a cell density of 1×10^5 cells/mL and incubated with sEVs, samples were at **1×10^{10} sEVs/mL (equivalent to 5×10^9 fluorescent sEVs/mL, ~25,000 sEVs fluorescent/cell)**, and incubations were terminated at **16 h**. Unlabeled sEVs served as negative controls to discriminate autofluorescence. Cells were gated for the main population using FSC-A vs SSC-A and doublets were excluded via FSC-A vs FSC-H. Fluorescent detection settings were: eGFP (488 nm laser; 525/40 nm filter), ACO-600™ (488 nm laser; 690/50 nm filter) and Cy5 (638 nm laser; 660/10 nm filter). Data was analyzed using CytExpert software provided with the cytometer. For flow cytometry performed with 3D culture models, the samples were analyzed on a BD Accuri C6 flow cytometer (BD Biosciences, Franklin Lakes, NJ, USA). sEVs were previously labelled with ACO-600™ to achieve a dose of **1.75×10^{10} ACO-600+ sEVs/mL, meaning ≈35,000 ACO-600+ sEVs/cell** and applied to the 3D cell model, incubations were carried out for 8 h. After incubation, apical and basolateral compartments were processed separately, first the apical layer was trypsinized to detach cells and further neutralizad with suplemented media**,** the basolateral compartment was then harvested and treated identically. Each cell suspension was washed twice with PBS by centrifugation at 1 500 rpm for 5 min, fixed with 4 % paraformaldehyde (PFA) for 30 min at RT, washed two more times with PBS (1 500 rpm, 5 min) and finally resuspended in 100 µL PBS. Unlabeled sEVs and untreated cell controls were included to define background and autofluorescence. Samples were stored at 4 °C and analyzed on the Accuri cytometer three days after preparation. Acquisition settings were adjusted to detect the ACO-600 signal using excitation at 640 nm and emission at 675/25 nm. Gating strategies were applied to exclude debris and doublets, ensuring accurate particle detection. Data were processed to quantify the percentage of ACO-600+ events within each gated compartment. All analyses were performed under identical instrument settings to allow direct comparison across samples.

CSFM (Leica TCS SP8 laser-scanning confocal spectral microscope, Leica Microsystems, Germany) was used to characterize fluorescent localization. It was performed using 24 well plate (SPL Life Sciences, 30024) adding 13 mm sterile glass coverslip (VWR, 631-0149) to each well and enough volume of gelatin from porcine skin 0,2 % (w/v) (Sigma Aldrich, G2500) to coat the coverslips for 30 min under UV light. Afterwards, gelatine was removed, and 2 mL of cells were seeded onto the coverslip at a cell density of 10^5 cell/mL in cell media. After 48 h, sEVs were added at 2.5×10^{10} sEVs/mL (≈50,000 sEVs/cell), meaning 1×10^{10} fluorescence sEVs/mL (≈25,000 fluorescent sEVs/cell). Incubation was carried out for 16 h and then prepared for observation. Between each of all following steps three washes with PBS were done. First, supernatant was removed, and cells were fixed for 20 min with formalin 10 % (v/v), then permeabilized with 0.1 % (v/v) Triton X-100 for 30 min, and samples were stained with 0.5 µg/mL DAPI (Sigma Aldrich, D9542) for 5 min. Finally, coverslips were mounted on the slides with a drop of Fluoromount™ mounting medium and visualized with the CSFM. Images were analyzed using ImageJ using identical parameters for all samples. The qualitative localization of fluorescence in the 3D co-culture model was assessed using a

Confocal Laser Scanning Microscope Leica TCS-SP5 AOBS, (Leica Microsystems, Germany). For uptake experiments, surface-modified sEVs were added to the apical compartment at a concentration of 2.5×10^{10} sEVs/mL (≈ 50,000 sEVs/cell), of which 1×10^{10} sEVs/mL corresponded to fluorescently labeled vesicles (≈ 25,000 fluorescent sEVs/cell), and cells were incubated for 8 h or 16 h. After incubation, both apical and basolateral compartments were fixed with 4% (v/v) PFA for 20 min, permeabilized with 0.1% (v/v) Triton X-100 for 10 min and washed twice with PBS between each step. The apical side was first stained with Phalloidin-647 (1/200) (Thermo Fisher Scientific, A22287) and DAPI (0.5 µg/mL) (Merck, D9542) in PBS (50 µL) for 30 min in the dark, followed by PBS washes, and the same staining procedure was then applied to the basolateral side. Finally, membranes were excised from the inserts, mounted in ibidi µ-Dishes (ibidi, 81158) with Fluoromount™ medium (Sigma F4680), and imaged by laser-scanning confocal microscopy. Z-stacks were acquired under identical settings for all samples, and image analysis was performed using ImageJ with consistent parameters across conditions.

Alternative uptake characterization was performed on an imaging flow cytometer (ImageStreamX, Cytek). For the experiment, surface-modified sEVs were added to the apical compartment of the 3D co-culture Transwell® model at 2.5×10^{10} sEVs/mL (≈ 50,000 sEVs/cell), of which 1×10^{10} sEVs/mL corresponded to fluorescently labeled vesicles (≈ 25,000 fluorescent sEVs/cell). Cells were incubated for 8 h. After incubation, apical and basolateral compartments were processed separately, the apical layer was trypsinized to detach cells, the reaction was neutralized with complete medium, and the basolateral compartment was then harvested and treated identically. Each cell suspension was washed twice with PBS by centrifugation at 1,500 rpm for 5 min, fixed with 4% (v/v) PFA for 30 min at room temperature, washed two times with PBS (1,500 rpm, 5 min), and finally resuspended in 100 µL PBS. Immediately before acquisition, suspensions were passed through a 40 µm mesh cell strainer (Corning, CLS431752) to remove aggregates. Unlabeled sEVs and untreated-cell controls were included to define background and autofluorescence. Samples were acquired using the 488 nm laser to detect eGFP with brightfield channels enabled for morphology and side scatter (SSC) collected to assess cellular granularity and refine singlet gating. Events were gated sequentially as follows: first, **in-focus** objects were selected by **Gradient RMS,** second, **single cells** were isolated by **Aspect Ratio vs Area,** third, **eGFP+** events were defined using a two-parameter gate on **Intensity vs Max Pixel.** A minimum of 60,000 events per sample were collected using the 40× objective at standard flow rate, with identical laser powers and camera gains across all conditions. eGFP+ and unlabeled controls were used to generate the compensation matrix and set fluorescence thresholds (n = 3). Data were analyzed in IDEAS® software. Results are reported as mean ± SD.

Explanation S3. QTRAP method for Lipid quantification.

Lipid quantification methodology was optimized by identifying the specific precursor and fragment ion masses characteristic of each phospholipid, ensuring selective and sensitive detection under QTRAP® conditions. Control experiments using unmodified vesicles were also performed, confirming that endogenous lipid species did not interfere with the selected transitions and that the recorded signals were specific to the inserted phospholipids [1]. Once the transitions were defined and validated, calibration curves were generated using known concentrations of each compound. Establishing these calibration curves allowing the conversion of raw signal intensities into absolute molecular quantities, thereby enabling precise calculation of the number of phospholipid molecules incorporated per vesicle.

An initial source of variability arose from the solvent system: when PBS was used, results showed high variability between replicates, most likely due to salt interference and ion suppression during electrospray ionization. To address this limitation, different solvents were tested, and methanol was selected as the optimal medium, since it improves solubility of amphiphilic lipids, reduces ionic interference, and provides more reproducible ionization, resulting in cleaner spectra and lower variability [2].

During ESI ionization, the long PEG (5 kDa) chain of DPA fragments extensively in-source, producing a variety of ions that make the intact molecule too large and heterogeneous to be reliably detected, and the complete DPA (5,812 Da) is larger than the maximum detectable size range of the instrument, making it impossible to visualize the intact precursor ion (**Figure SE.3.1.A**). For this reason, the analysis was focused on the DSPE lipid anchor, which is much smaller and generates stable fragments.

The intact lipid anchor $[M+H]^+$ was detected as a precursor ion at *m/z* 749.1, corresponding to the distearoyl phosphatidylethanolamine backbone (two stearoyl chains, phosphate, and ethanolamine headgroup). Upon low-energy collision-induced dissociation (CID), fragmentation occurred at the phosphoethanolamine headgroup, specifically through the loss of a methylene unit 14 Da ($-CH_2-$), resulting in a stable product ion at *m/z* 735 (**Figure SE.3.1.B.**). This transition (*m/z* 749.1 to 735) is consistent with well-described fragmentation pathways of phosphatidylethanolamines (PE) and was selected for the quantification of DPA incorporated into the sEV membranes, for which the corresponding calibration curve was generated (**Figure SE.3.1.C**.) [3–5].

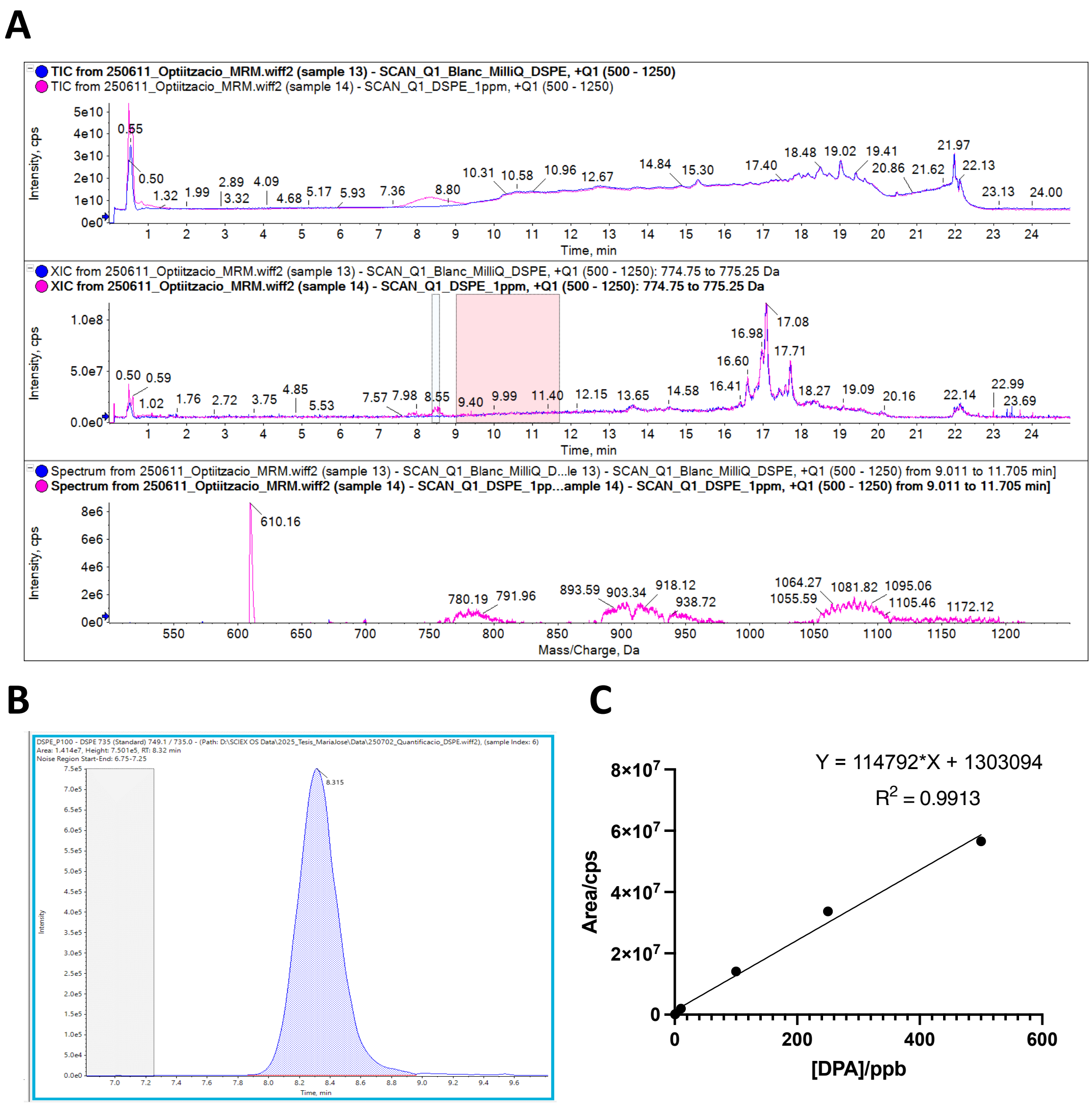

***Figure SE.3.1.: A**) QTRAP® mass spectrometry analysis of DSPE-PEG(5000)-Azide (DPA) showing the extracted ion chromatogram and the selected MRM transition (precursor* m/z *749.1 to product* m/z *735). (**B**) Representative peak area of the selected transition. (**C**) Calibration curve obtained from standard solutions.*

Detection of DLPC (621.82 Da), was more straightforward than for DPA, since the molecule falls within the mass range of the instrument and produces a well-defined fragmentation profile. The precursor ion selected was *m/z* 622.9, corresponding to the protonated $[M+H]^+$ form of 1,2-dilauroyl-sn-glycero-3-phosphocholine (**Figure SE.3.2.A.**). Upon CID, a characteristic product ion at *m/z* 184.0 was obtained (**Figure SE.3.2.B.**), which corresponds to the phosphocholine headgroup ($C_5H_{15}NO_4P^+$). This fragment is a well-known diagnostic ion for phosphatidylcholines, widely used in lipidomics because of its high intensity and specificity, and therefore it was chosen as the marker for DLPC

quantification[6]. Based on this transition, the corresponding calibration curve was generated (**Figure SE.3.2.A.**).

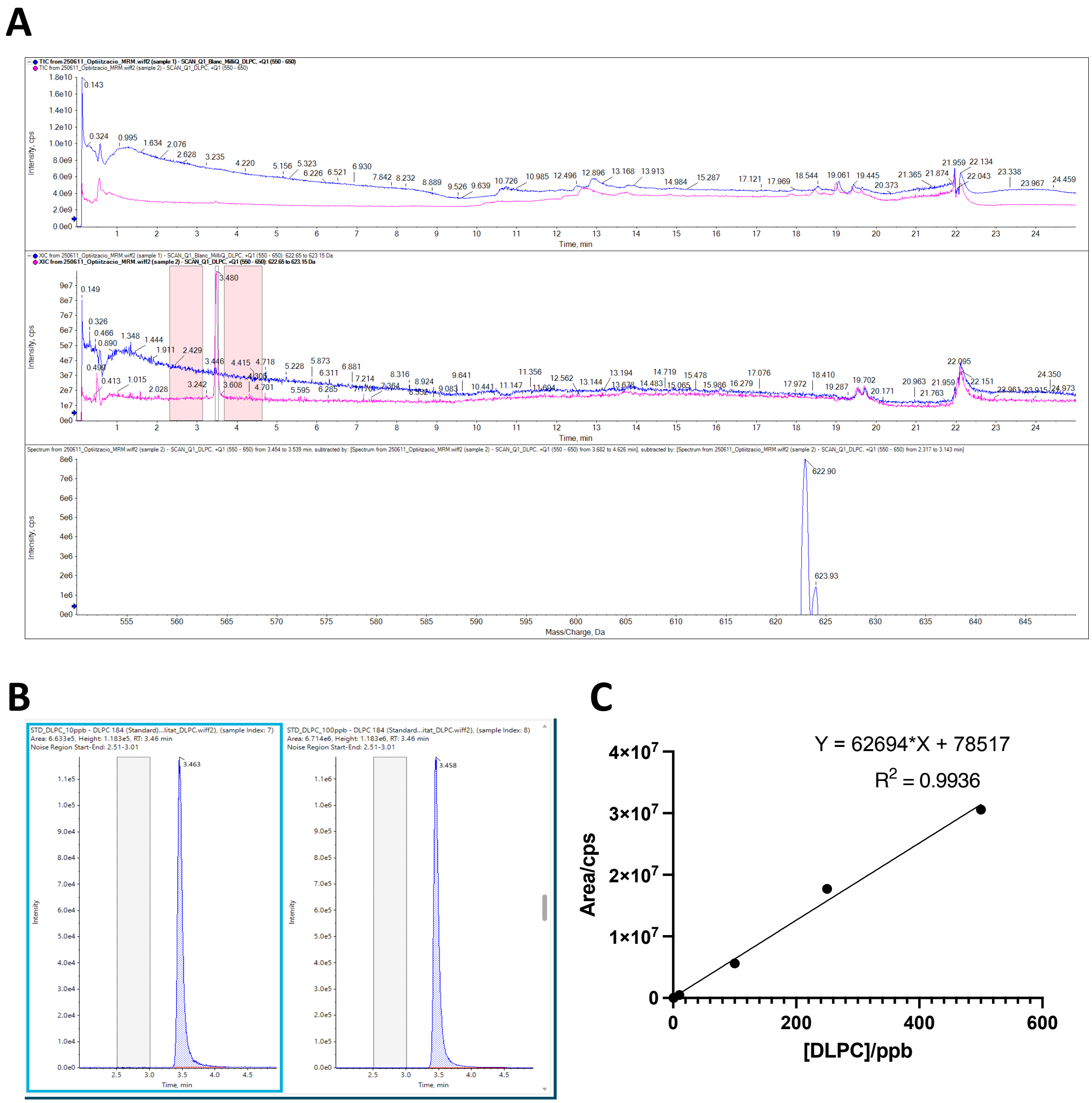

Figure SE.3.2. A*) QTRAP® mass spectrometry analysis of DLPC showing the extracted ion chromatogram and the selected MRM transition (precursor* m/z *622.9 to product* m/z *184). (****B****) Representative peak area of the selected transition. (****C****) Calibration curve obtained from standard solutions.*

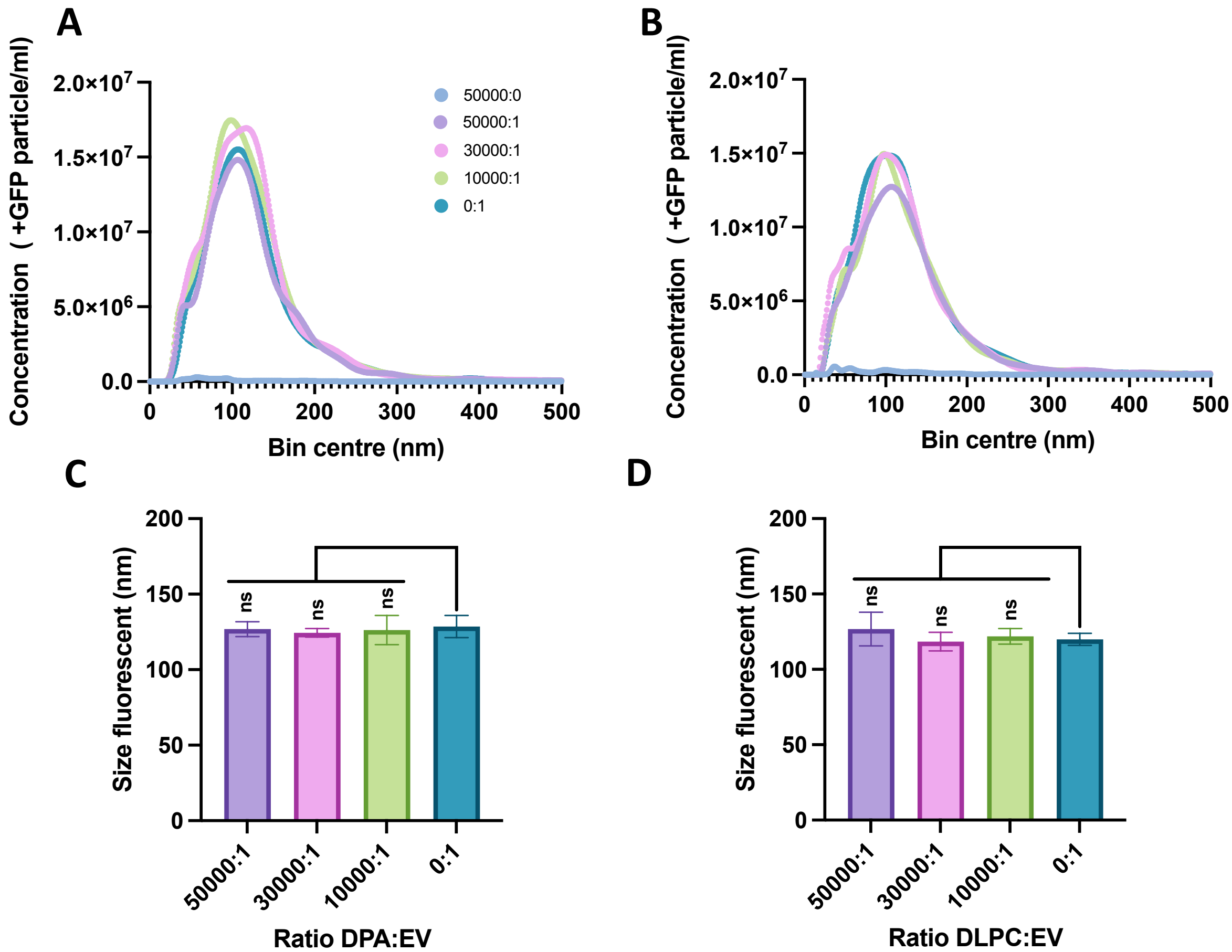

Figure S1: Physicochemical characterization of +eGFP sEVs modified with different of DPA:EV or DLPC:EV ratios by nanoparticle tracking analysis (NTA). *(**A**) Particle concentration of DPA-modified + eGFP sEVs. (**B**) Particle concentration of DLPC-modified+ eGFP sEVs. (**C**) Particle size mean of DPA-modified+ eGFP sEVs. (**D**) Particle size mean of DLPC-modified+ eGFP sEVs. Data are presented as mean ± SD (n = 3). Statistical analysis: ns = not significant.*

With fluorescent NTA mode (sEVs with GFP-CD81) the concentration vs size distributions overlapped across all lipid ratios for DPA (**Figure S1.A.)** and DLPC (**Figure S1.B.**), with a peak around 120-140 nm, indicating that the abundance and number-based size distribution of labeled sEVs remained stable after lipid insertion. As expected, overall particle concentrations were lower than in non-fluorescent mode because only a fraction of the vesicle population is fluorescently labeled, specifically, only sEVs incorporating enough molecules of the eGFP-CD81 fusion protein are detected. The 50,000:0 control was essentially at baseline because it contained no eGFP+ sEVs, any structures formed by phospholipids lacked fluorescence and were therefore not detected. Regarding mean size, the bar plots for DPA (**Figure S1.C.**) and DLPC (**Figure S1.D.**) showed no significant

differences compared with the 0:1 control, indicating that DPA or DLPC insertion did not alter the size of sEVs detected by NTA.

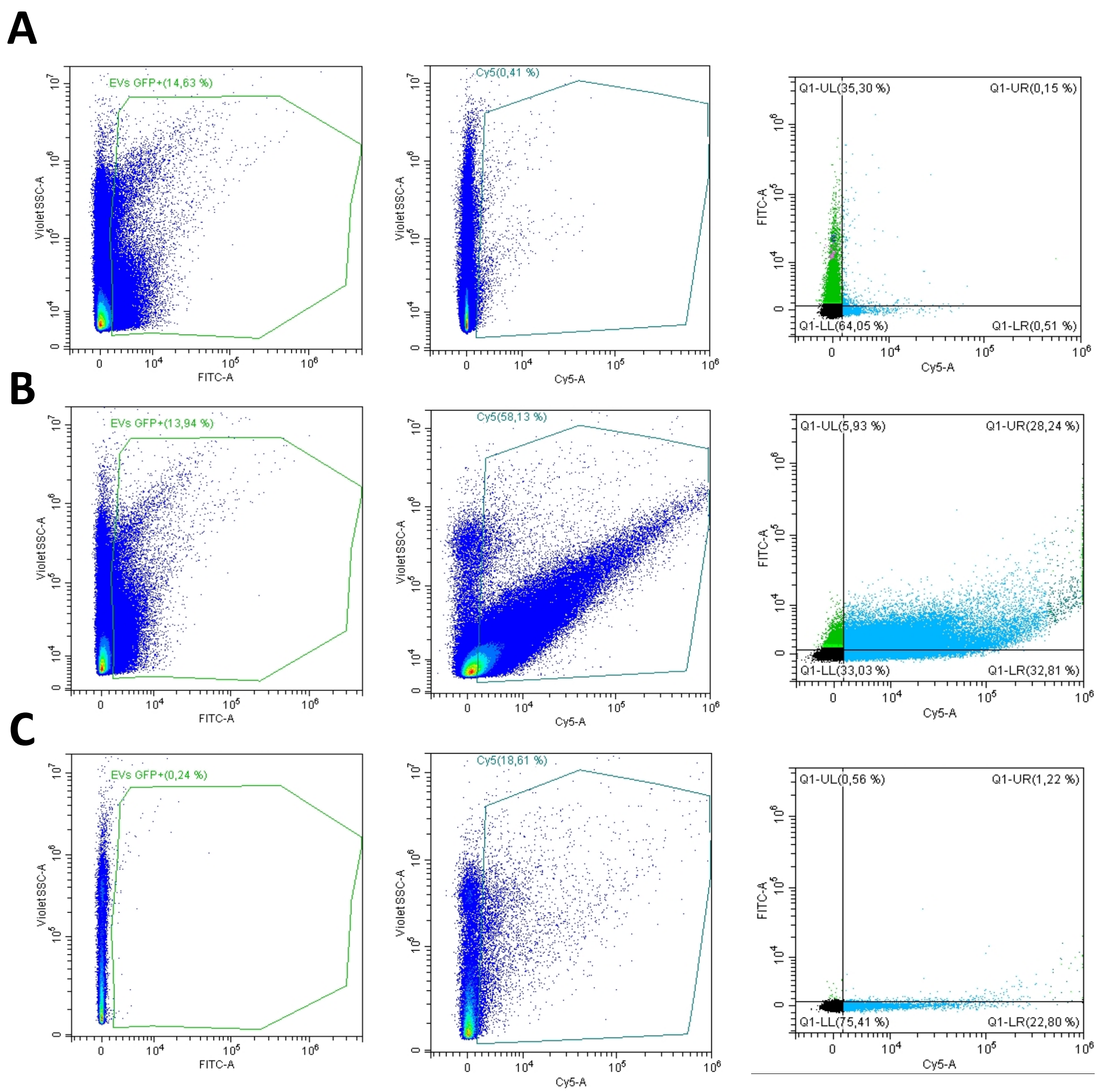

Figure S2: Representative plots from nano-flow cytometry analysis of sEVs after labeling with DPA-Cy5. *The first column shows Violet SSC signal versus eGFP fluorescence, the second column shows Violet SSC signal versus Cy5 fluorescence, and the third column displays GFP versus Cy5 fluorescence for dual labeling. (**A**) Negative control (0:1, eGFP unmodified vesicles), (**B**) vesicles modified with DPA-Cy5 at 50,000:1 ratio, and (**C**) phospholipid-only control (50,000:0).*

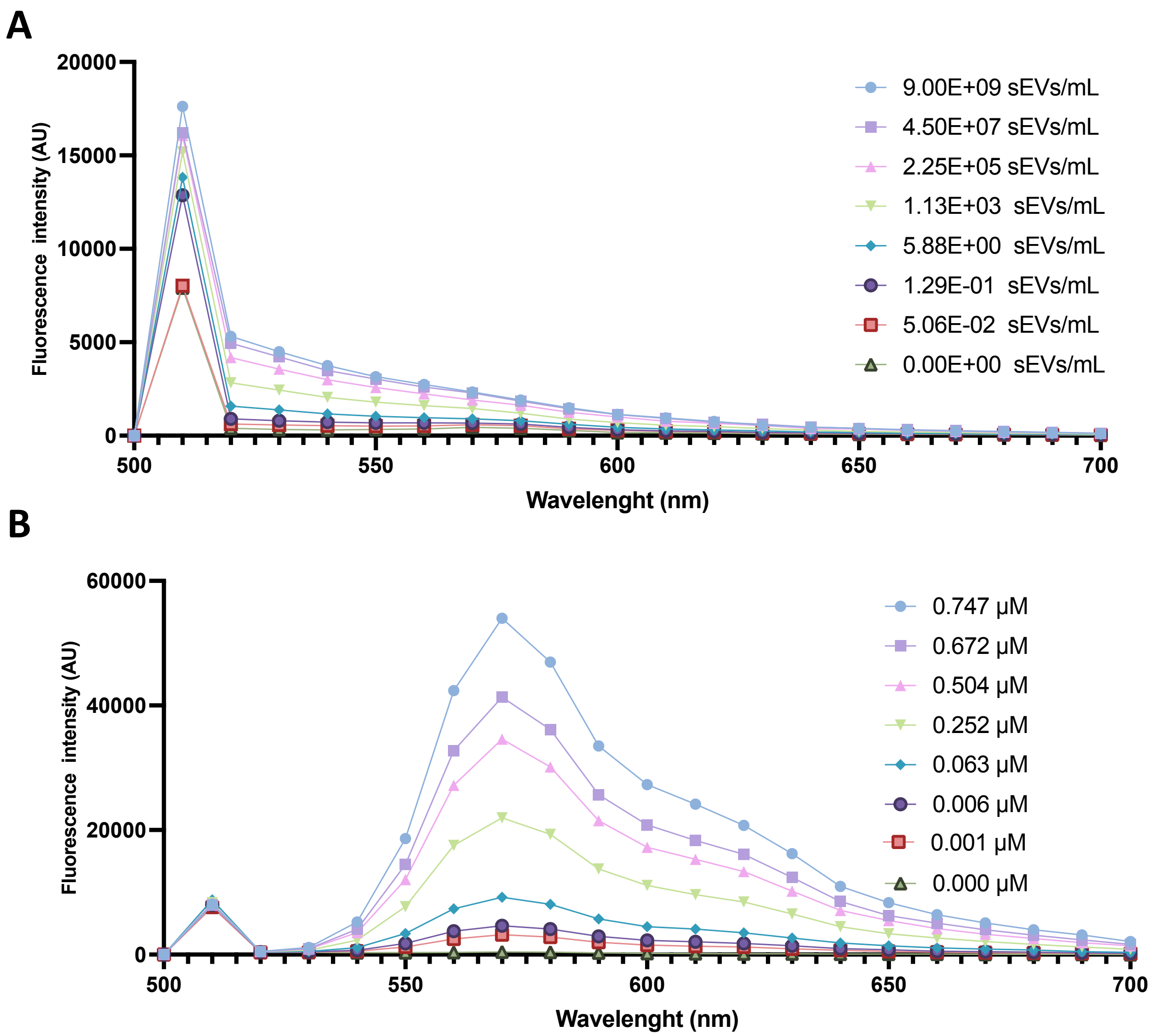

Figure S3: Fluorescence emission spectra used to determine optimal excitation/emission wavelengths for FRET measurements. *(**A**) Emission spectra of eGFP expressed in sEVs at different vesicle concentrations. (**B**) Emission spectra of DPA-Cy3 at different molar concentrations.*

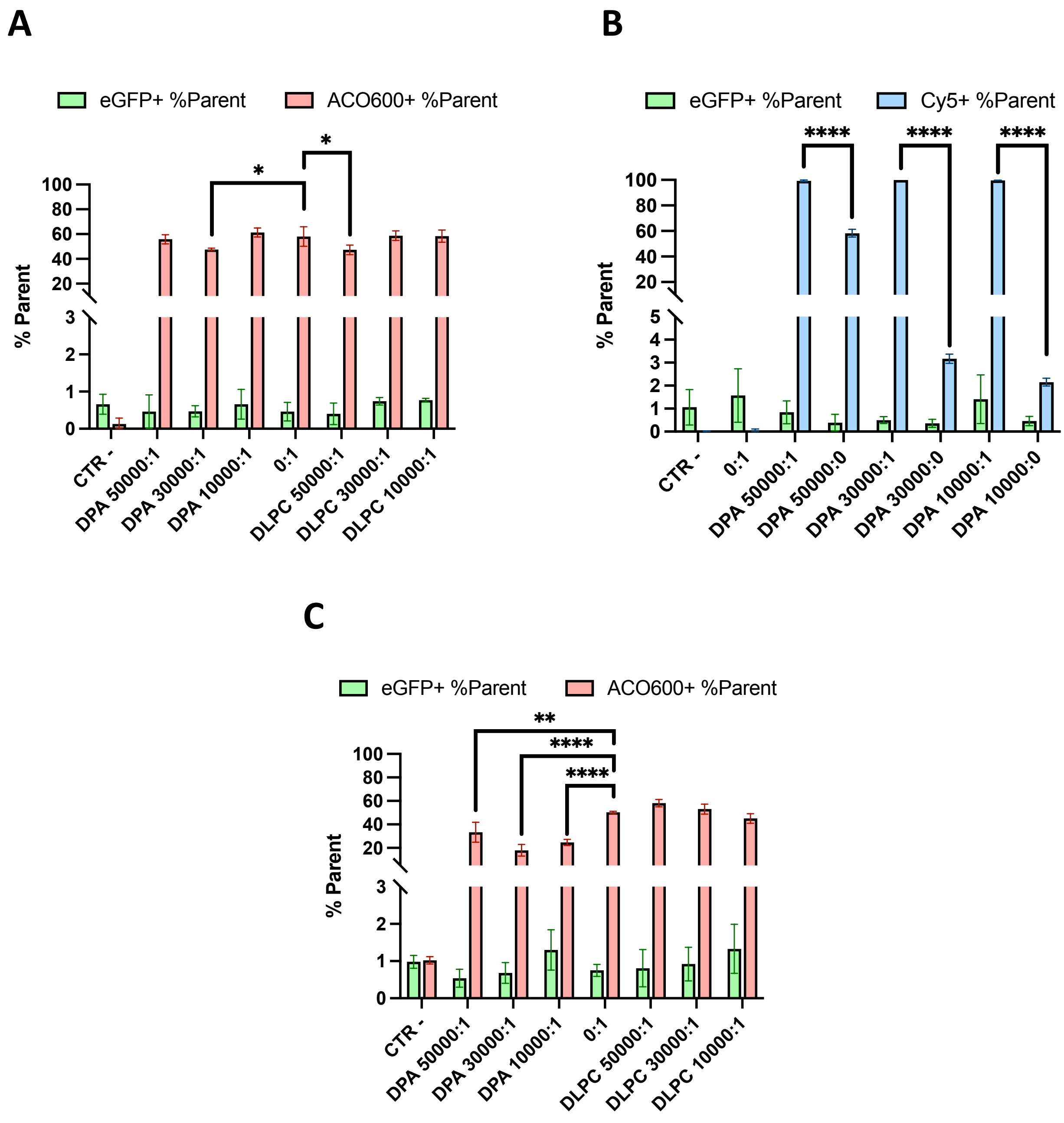

Figure S4: Flow cytometry analysis of cellular uptake of eGFP surface-modified sEVs. *(A) Uptake in parental HEK293 cells quantified with ACO600 dye. (B) Internalization of free phospholipid controls tracked with DPA-Cy5. (C) Uptake in BEAS-2B pulmonary epithelial cells. Statistical analysis: ns = not significant, ****p < 0.0001, ***p < 0.001, **p < 0.01 and *p < 0.1.*

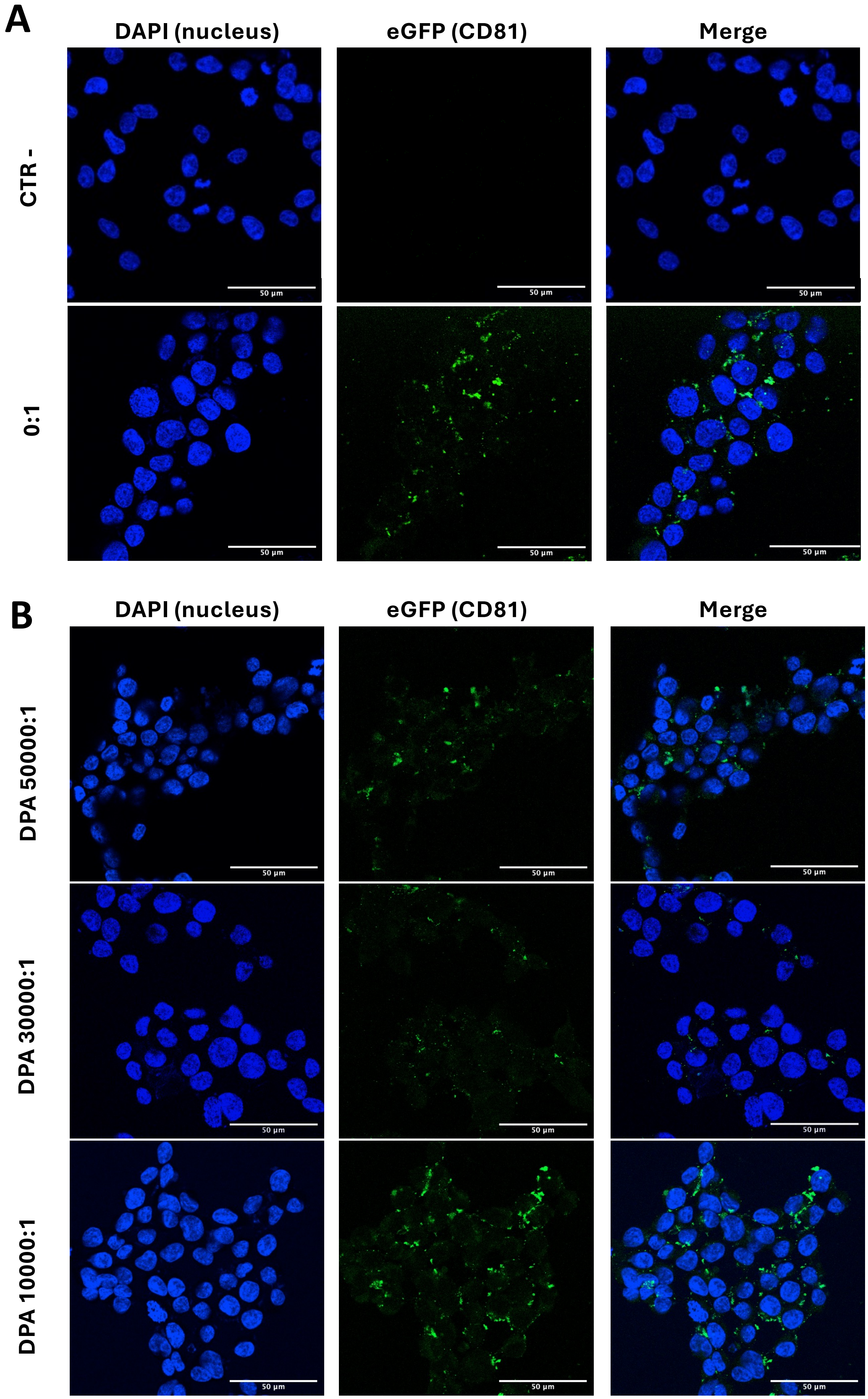
A
DAPI (nucleus)
eGFP (CD81)
Merge
CTR -
0:1
50 µm
B
DAPI (nucleus)
eGFP (CD81)
Merge
DPA 50000:1
DPA 30000:1
DPA 10000:1
50 µm

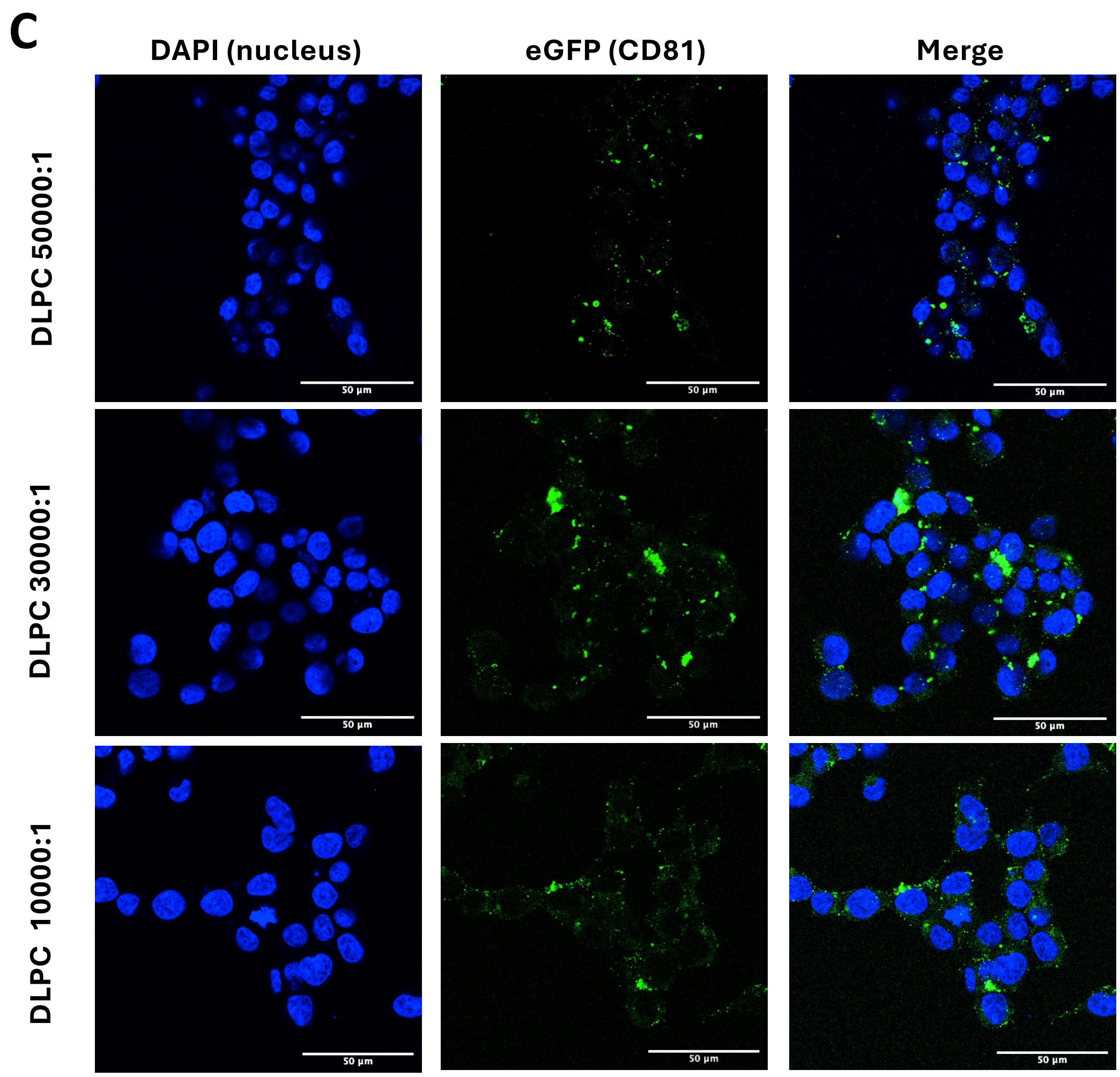

Figure S5: Confocal microscopy analysis of sEVs uptake in HEK293 cells. *Nuclei were stained with DAPI (blue), while eGFP-CD81 expressed in sEVs provided the green signal. (**A**) Control (CTR -) and unmodified vesicles (0:1). (**B**) DPA-modified vesicles at different lipid-to-vesicle ratios (50,000:1, 30,000:1, 10,000:1). (**C**) DLPC-modified vesicles at corresponding ratios (50,000:1, 30,000:1, 10,000:1). Images were acquired using a 40× objective with 2× zoom. Scale bar: 50 µm.*

Figure S5. A. shows the control condition (CTR -) and unmodified sEVs (0:1), where internalized vesicles are clearly visualized as green dots. **Figure S5.B.** corresponds to DPA-modified sEVs at different lipid-to-vesicle ratios, whereas **Figure S5.C.** shows the results obtained with DLPC-modified sEVs. Across the different conditions, eGFP dots were visible for both unmodified and lipid-modified sEVs, indicating that DPA- and DLPC-engineered vesicles retain the capacity to interact with and be internalized by cells. However, the fluorescence signal appeared more pronounced in certain conditions, particularly DPA 10,000:1 and DLPC 30,000:1, which correspond to ratios previously

identified as optimal for stable lipid incorporation without extensive aggregation. By contrast, at DPA 30,000:1, the fluorescence appeared less intense, which may reflect a partial shielding effect of the PEGylated lipids at this ratio, slightly reducing uptake efficiency. Similarly, higher lipid ratios such as DPA 50,000:1 or DLPC 50,000:1, although still showing eGFP spots, appeared less intense overall, consistent with a partial reduction of uptake due to excessive surface coating.

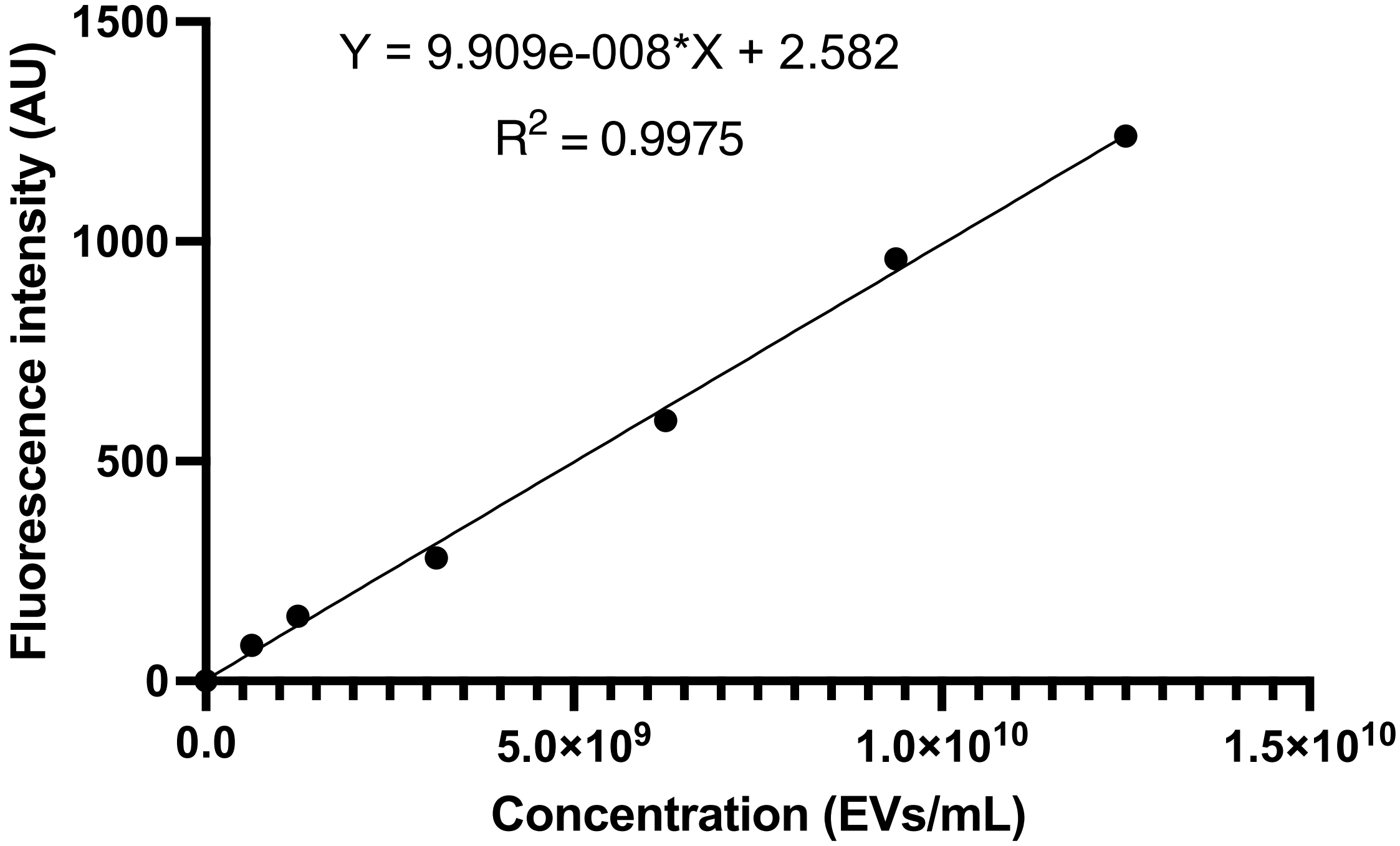

Figure S6: Linear correlation between fluorescence intensity (AU) and concentration of fluorescent (EVs/mL) and equation used for mucin permeability calculations.

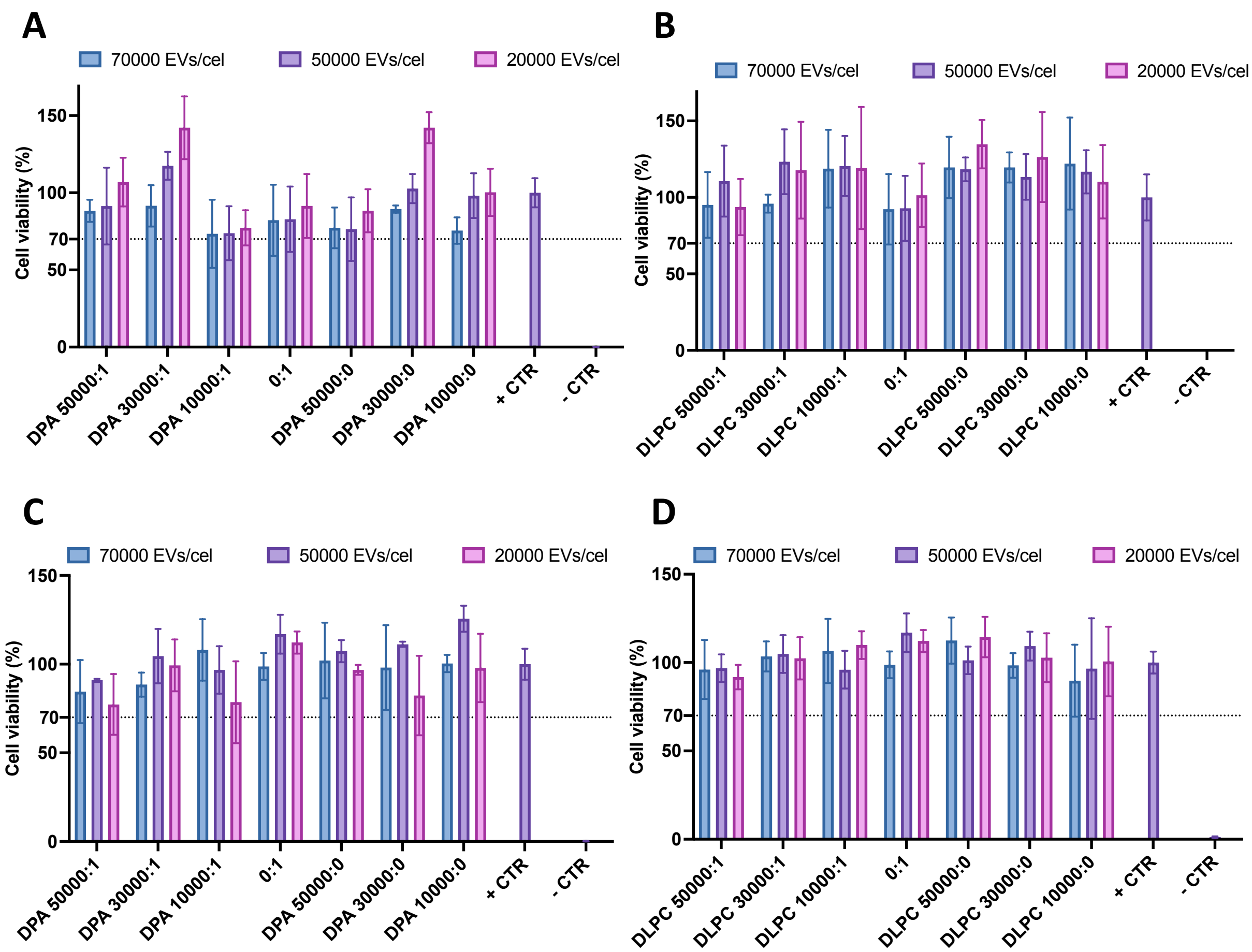

Figure S7: Cytotoxicity of free phospholipids, unmodified sEVs, and surface-modified sEVs in pulmonary epithelial and endothelial cell. *(**A**) DPA-modified sEVs and free DPA phospholipids in NCI-H441 cells. (**B**) DLPC-modified sEVs and free DLPC phospholipids in NCI-H441 cells. (**C**) DPA-modified sEVs and free DPA phospholipids in HPMEC-ST1.6R cells. (**D**) DLPC-modified sEVs and free DLPC phospholipids in HPMEC-ST1.6R cells.* Three EV concentrations (20,000, 50,000, and 70,000 EVs/cell) were tested, shown in pink, purple, and dark blue, respectively. The dashed line represents the 70% viability threshold. Data are presented as mean ± SD (n = 3).

Before evaluating the transport of surface-modified sEVs with the cells used pulmonary 3D cell barrier, it was essential to confirm that both the vesicles and the lipid modifications did not compromise cell viability. For this purpose, a resazurin cytotoxicity assay was performed. In this assay, the potential cytotoxicity of the individual phospholipids (DPA and DLPC), unmodified sEVs, and the complete surface-modified sEVs formulations at different concentrations was assessed simultaneously. This experimental design enabled a direct comparison between the samples under identical conditions. Needed for confirming that none of the tested concentrations compromised cell viability, this step provides a critical link between the design of the formulations and their application in the 3D pulmonary cell barrier, ensuring that all subsequent experiments were conducted

under non-toxic conditions. Cell viability was assessed in both epithelial (NCI-H441) and endothelial (HPMEC-ST1.6R) cells after exposure to phospholipids surface-modified sEVs at different concentrations (20,000, 50,000, and 70,000 EVs/cell).

In NCI-H441 cells, DPA-modified vesicles (**Figure S7.A.**) showed a tendency toward slightly reduced viability at the highest vesicle dose (70,000 EVs/cell), but no concentration dropped below 70%. Free DPA at equivalent phospholipid concentrations (50,000:0, 30,000:0, 10,000:0) showed comparable results, indicating that the phospholipid alone did not induce additional cytotoxicity. For DLPC (**Figure 7.B.**), a similar pattern was observed, with all conditions maintaining high viability and no clear dose-dependent toxicity. In HPMEC cells, for all conditions including the phospholipids alone, DPA-modified vesicles (**Figure 7.C.**) and DLPC-modified vesicles (**Figure 7.D.**) cell viability remained above 70% at all doses (20,000, 50,000, and 70,000 EVs/cell), with no clear dose-response pattern. The variations observed fall within the experimental variability, and no consistent differences were detected between free phospholipids and surface-modified vesicles. Overall, the results indicate no detectable cytotoxicity in HPMEC cells under the tested conditions.

Overall, these results indicate that both DPA and DLPC, whether tested as free phospholipids or incorporated into sEV formulations, did not compromise the viability of epithelial (NCI-H441) or endothelial (HPMEC-ST1.6R) cells, with values consistently above 70%. This lack of cytotoxicity is consistent with the intrinsic biocompatibility of the tested components, sEVs are membrane-derived nanovesicles with low toxicity, DLPC mimics endogenous phosphatidylcholine lipids, and DPA (PEGylated lipid) forms a hydrated corona that minimizes nonspecific interactions. Moreover, the concentrations tested are below levels typically associated with membrane destabilization, which explains the absence of a dose-dependent decrease in viability. The resazurin assay further showed no differences between free lipids and lipid-modified vesicles, supporting the safety of the formulations. Nevertheless, these results should be confirmed through repeated experiments to fully validate the absence of cytotoxic effects.

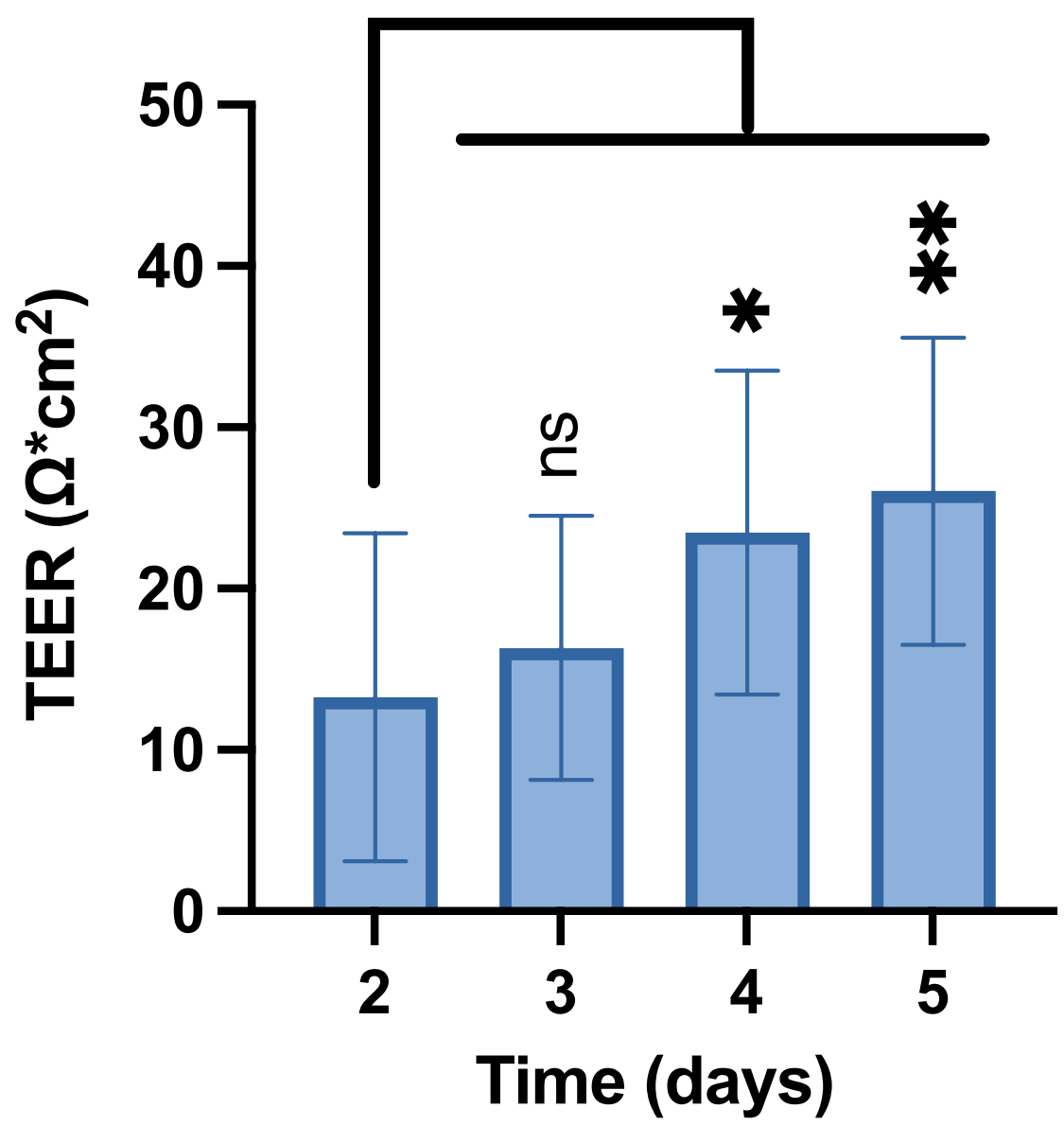

Figure S8: **Establishment and validation of the 3D pulmonary cell barrier model.** *Transepithelial electrical resistance (TEER) values measured over 5 days of culture. Data are presented as mean ± SD (n=3). Statistical analysis was performed by one-way ANOVA followed by Tukey's multiple comparison test (*p < 0.05; **p < 0.01 ns = not significant).*

To ensure that the barrier developed correctly and maintained integrity before performing permeability assays, transepithelial electrical resistance (TEER) was monitored. TEER provides a non-invasive measurement of tight junction formation and paracellular permeability, serving as a reliable control of barrier quality and reproducibility. **Figure** presents TEER values measured over the 5-day culture period. A progressive increase in resistance was observed, reaching approximately 25 Ω·cm² on day 5, the final day of culture. This increase reflects the maturation of tight junctions and the establishment of a functional barrier. Importantly, these values are consistent with those previously reported as indicative of correct barrier formation [7–9], confirming that only inserts with the expected integrity were selected for subsequent permeability experiments with sEVs.

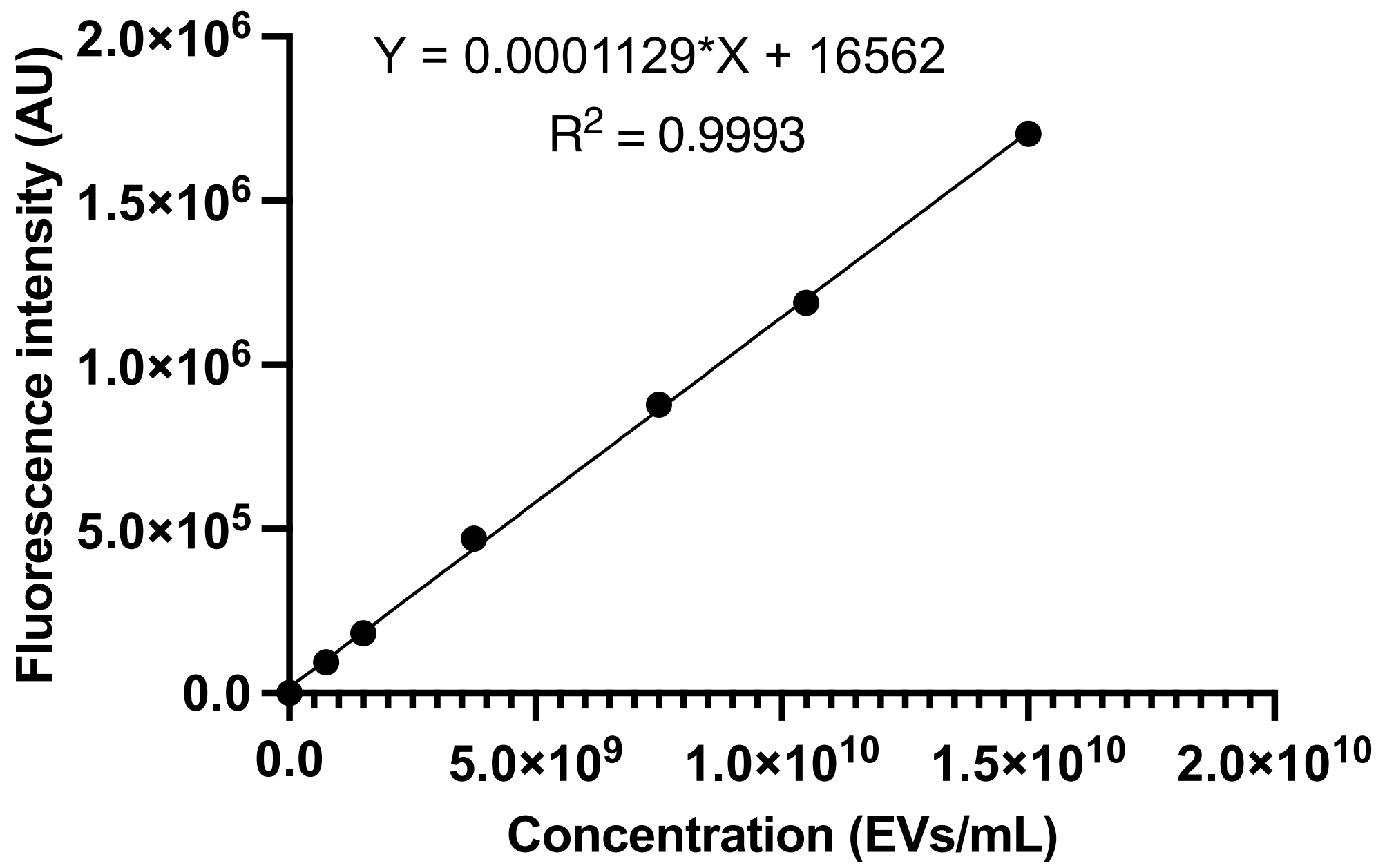

***Figure S9**: Linear correlation between fluorescence intensity (AU) and concentration of fluorescent sEVs (EVs/mL) and equation used for 3D pulmonary model permeability calculations.*

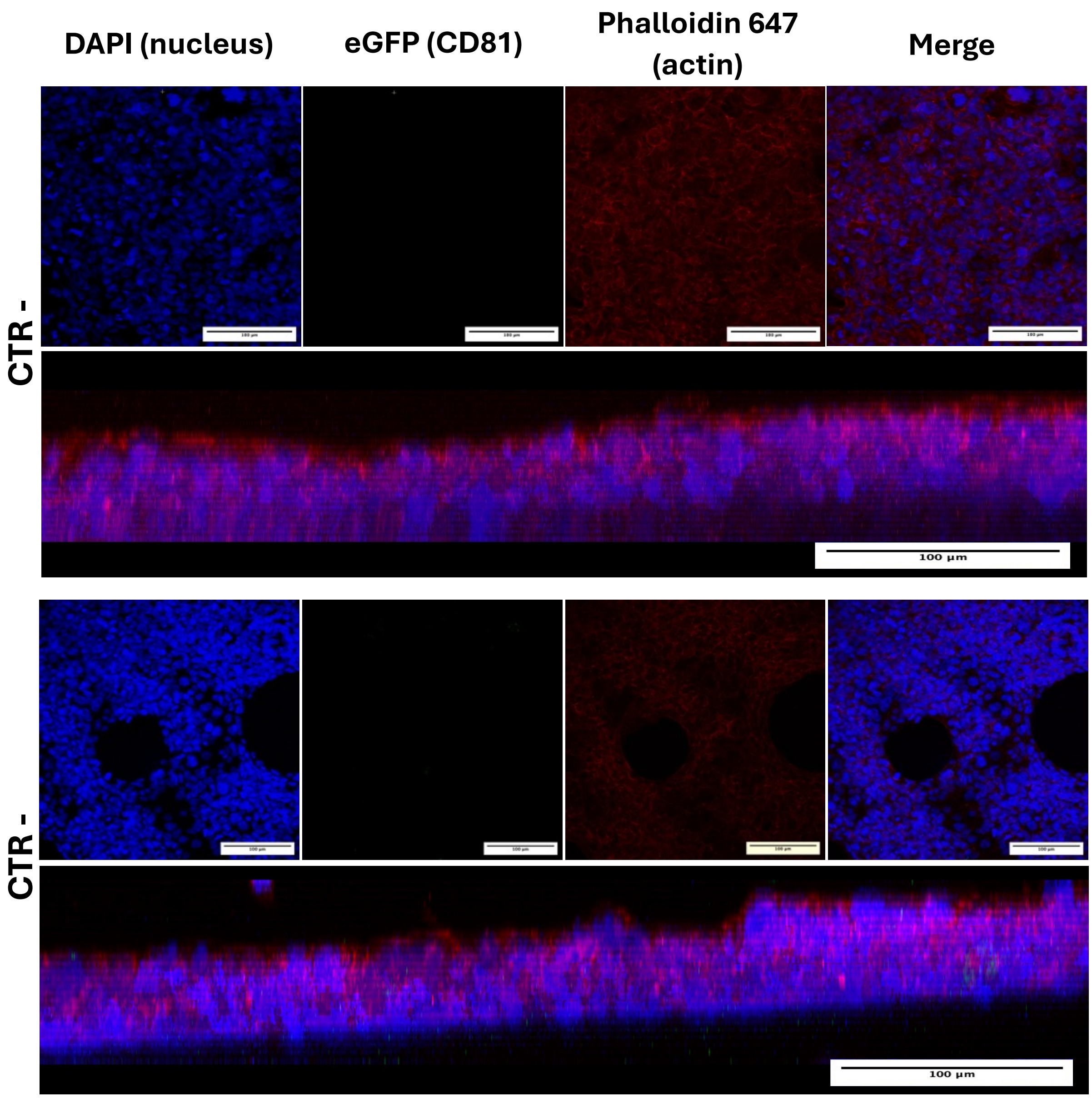

Figure S10: Confocal microscopy analysis of a control 3D pulmonary barrier model.

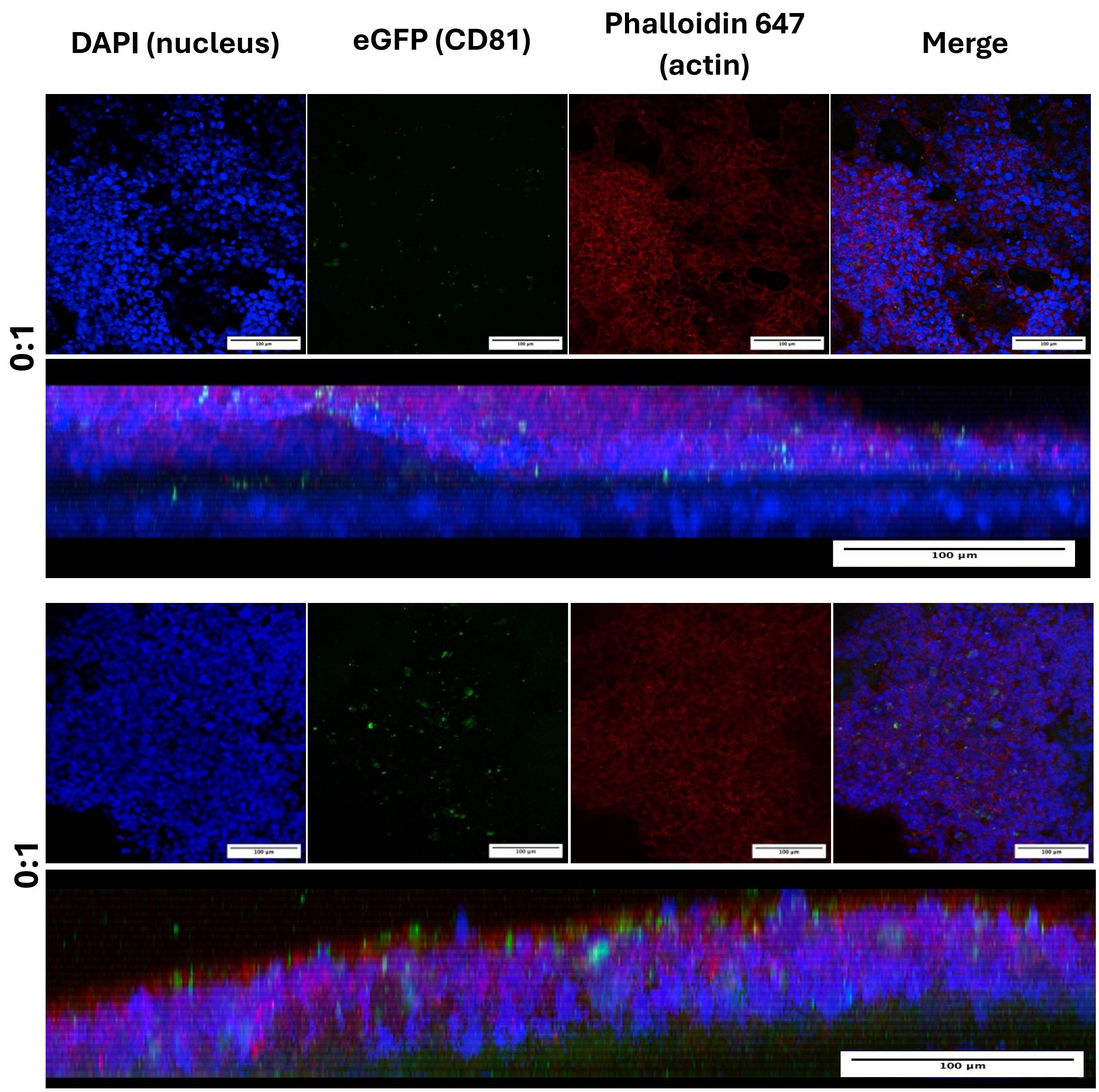

Figure S11: Confocal microscopy analysis of non-modified EVs incubated on a 3D pulmonary barrier model.

ADDITIONAL REFERENCES

1. Vilela, A. F. L. *et al.* Optimizing Lipidomics Analysis Workflows for Biological Fluids and Extracellular Vesicles with Integrated Liquid Chromatography Tandem Mass Spectrometry Approaches. *BioRxiv* Preprint at https://doi.org/10.1101/2024.11.08.622653 (2024).

2. Sitnikov, D. G., Monnin, C. S. & Vuckovic, D. Systematic Assessment of Seven Solvent and Solid-Phase Extraction Methods for Metabolomics Analysis of Human Plasma by LC-MS. *Scientific Reports 2016 6:1* **6**, 1–11 (2016).

3. Kiyonami, R. *et al. Characterization and Quantification of Lipid Nanoparticle Components and Their Degradants in Vivo Using an LC-HRAM MS Platform*. *ASTM* (2022) doi:10.1520/E3324-22.

4. Pi, J., Wu, X. & Feng, Y. Fragmentation patterns of five types of phospholipids by ultra-high-performance liquid chromatography electrospray ionization quadrupole time-of-flight tandem mass spectrometry. *Analytical Methods* **8**, 1319–1332 (2016).

5. Majumdar, S., Nasini, U. B. & Patri, A. K. A Standard Test Method for Lipid Quantitation in Liposomal Formulations Using Ultra-High-Performance Liquid Chromatography with Triple Quadrupole Mass Spectrometry (UHPLC-TQMS). *ASTM International, West Conshohocken, PA, USA* https://doi.org/10.1520/E3324-22 (2022) doi:10.1520/E3324-22.

6. Ekroos, K. *et al.* Charting molecular composition of phosphatidylcholines by fatty acid scanning and ion trap MS3 fragmentation. *J. Lipid Res.* **44**, 2181–2192 (2003).

7. Barros, A. S., Costa, A. & Sarmento, B. Building three-dimensional lung models for studying pharmacokinetics of inhaled drugs. *Advanced Drug Delivery Reviews* vol. 170 386–395 Preprint at https://doi.org/10.1016/j.addr.2020.09.008 (2021).

8. Hameedat, F., Pinto, S., Marques, J., Dias, S. & Sarmento, B. Functionalized zein nanoparticles targeting neonatal Fc receptor to enhance lung absorption of peptides. *Drug Deliv. Transl. Res.* **13**, 1699–1715 (2023).

9. Costa, A., de Souza Carvalho-Wodarz, C., Seabra, V., Sarmento, B. & Lehr, C. M. Triple co-culture of human alveolar epithelium, endothelium and macrophages for studying the interaction of nanocarriers with the air-blood barrier. *Acta Biomater.* **91**, 235–247 (2019).
